# Flexibility of Poly(alkyl methacrylate)s Characterized by Their Persistence Length Determined through Pyrene Excimer Formation

**DOI:** 10.3390/polym16152126

**Published:** 2024-07-26

**Authors:** Kristijan Lulic, Grégoire Muller, Renzo Gutierrez, Hunter Little, Jean Duhamel

**Affiliations:** Institute for Polymer Research, Waterloo Institute for Nanotechnology, Department of Chemistry, University of Waterloo, 200 University Avenue West, Waterloo, ON N2L 3G1, Canada; klulic@uwaterloo.ca (K.L.); gregoire.muller2@etu.unistra.fr (G.M.); renzo.gutierrez@uwaterloo.ca (R.G.); htlittle@uwaterloo.ca (H.L.)

**Keywords:** pyrene excimer formation, persistence length, poly(alkyl methacrylate)

## Abstract

A series of poly(alkyl methacrylate)s and poly(oligo(ethylene glycol) methyl ether methacrylate)s labeled with 1-pyrenebutanol were referred to as the PyC_4_-PC_n_MA samples with *n* = 1, 4, 6, 8, 12, and 18 and the PyC_4_-PEG_n_MA samples with *n* = 0–5, 9, 16, and 19, respectively. Pyrene excimer formation (PEF) upon the encounter between an excited and a ground-state pyrenyl labels was employed to determine their persistence length (*l*_p_) in *o*-xylene. The fluorescence decays of the PyC_4_-PC_n_MA and PyC_4_-PEG_n_MA samples were acquired and analyzed with the fluorescence blob model to yield the number (*N*_blob_) of structural units in the volume probed by an excited pyrenyl label. *N*_blob_ was found to decrease with an increasing number (*N*_S_) of non-hydrogen atoms in the side chain, reaching a plateau for the PyC_4_-PEG_n_MA samples with a longer side chain (*n* = 16 and 19). The *N*_blob_ values were used to determine *l*_p_. The *l*_p_ values for the PyC_4_-PC_n_MA and PyC_4_-PEG_n_MA samples increased linearly with increasing *N*_S_^2^ as predicted theoretically, which agreed with the *l*_p_ values obtained by viscometry for a series of PC_n_MA samples. The good agreement between the *l*_p_ values retrieved by PEF and viscometry served to validate the PEF-based methodology for determining *l*_p_ for linear polymers.

## 1. Introduction

Backbone flexibility represents an important parameter in the characterization of a polymer. It affects its glass transition, which defines whether a plastic will be used as a hard (brittle) plastic or a rubbery elastomer at temperatures below or above its glass transition temperature (*T*_g_), respectively [1]. More specifically, a stiffer backbone like that of polystyrene yields a higher glass transition temperature (*T*_g_) of +100 °C [2] compared to that of a polymer with a more flexible backbone like poly(ethylene oxide) with a *T*_g_ of −53 °C [3,4]. Furthermore, more flexible polymeric backbones enable more contacts and interactions between the structural units (SU) constituting the polymer. This matters for polypeptides like proteins where the flexibility of the polypeptidic backbone promotes interactions between amino acids, which lead to the formation of specific secondary structures, eventually leading to the folded protein. For instance, more flexible polypeptides containing glycine [5] and alanine [6] generate more contacts between amino acids, which results in longer protein folding times [7]. Another property affected by polymer backbone flexibility is the stiffness of the resulting polymeric material defined by its modulus. Direct relationships between the modulus of a polymeric material and the persistence length (*l*_p_)—a physical measure of polymer backbone flexibility—have been proposed theoretically [8,9,10] and observed experimentally for semi-rigid linear chains like polymeric bottlebrushes [11] and DNA [12]. Backbone flexibility can also be taken advantage of to control the expansion of a polymer coil upon increasing the solution temperature or adjusting its contour length as it interacts with the surface of wax crystals, which is being done with the poly(alkyl methacrylate)s (PC_n_MA) used, as viscosity index improvers (VII) or pour point depressants (PPD), respectively, by the oil additive industry [13]. These examples illustrate the importance of polymer backbone flexibility and how it impacts polymer properties for diverse applications.

Polymer backbone flexibility can be described by the persistence length (*l*_p_), which represents the distance between a reference point and a secondary point taken along the contour length (*L*_c_) of the polymer chain, where the vector tangent to the chain at the secondary point has lost its orientation with respect to the original orientation of the vector tangent to *L*_c_ at the reference point. Traditionally, *l*_p_ has been determined through the analysis of conformational plots generated by measuring the intrinsic viscosity ([*η*]) by viscometry [14] or the radius of gyration (*R*_G_) by scattering techniques [15,16] for a series of polymer samples prepared with a narrow molecular weight distribution (MWD) and dissolved in a θ-solvent. Unfortunately, many polymers cannot be prepared with a narrow MWD. For these polydisperse polymer samples, a gel permeation chromatography (GPC) instrument equipped with a light scattering or viscosity detector can be used to obtain [*η*] or *R*_G_ as a function of molecular weight, which can then be used to build the corresponding conformational plots [17,18]. Unfortunately, GPC instruments require careful calibration and are typically operated with the same solvent in which the polymers of interest might not be soluble, and the polymers might not interact with the packing material of the GPC columns, so that the GPC trace truly reflects the MWD of the polymer sample.

The difficulties encountered in accommodating all these experimental requirements rationalize why *l*_p_ remains unknown for many polymer backbones and justify the search and development of methodologies based on techniques other than viscometry, scattering, and GPC to determine *l*_p_. One such methodology is based on pyrene excimer formation (PEF) between excited and ground-state pyrenyl labels, which are randomly and covalently attached onto a polymer [19,20]. Pyrene was selected over other excimer-forming fluorophores like naphthalene or perylene for the following reasons. The four aromatic rings of pyrene enable much more efficient excimer formation than naphthalene with only two aromatic rings [21], and the fluorescence of the pyrene monomer and excimer are well-resolved, allowing their separate detection in the fluorescence spectrum, which is not the case for perylene with its five aromatic rings [22]. 

**Figure 1:**
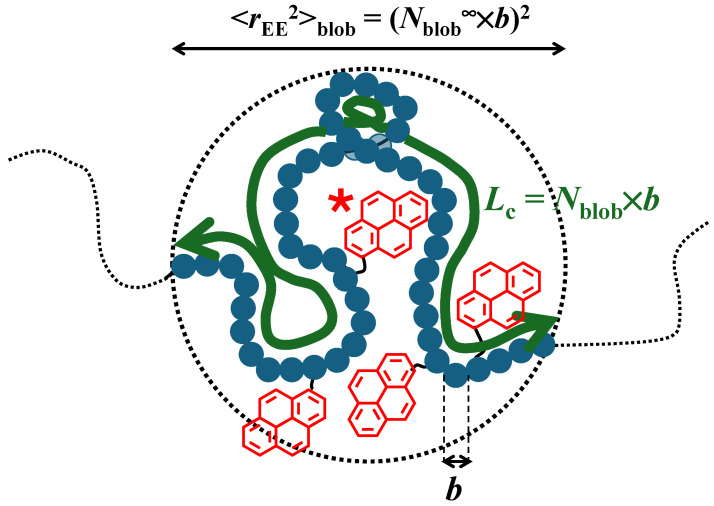
Schematic representation of a *blob* made of *N*_blob_ structural units represented as blue beads of length *b* with a contour length *L*_c_ equal to *N*_blob_ × *b* and a squared end-to-end distance <*r*_EE_^2^> equal to (*N*_blob_^∞^ × *b*)^2^. The excited pyrenyl label is indicated by a star.

The methodology based on PEF takes advantage of the fact that since a pyrenyl label attached to a polymer chain remains excited for a finite and short period of time (<1 μs), it can only probe a volume referred to as a *blob*, which is much smaller than the polymer coil. Since each pyrenyl label probes the same *blob*, the polymer coil can be divided into a cluster of identical *blobs*, among which the randomly attached pyrenyl labels distribute themselves according to a Poisson distribution. The fluorescence *blob* model (FBM) can then be applied to fit the fluorescence decays acquired with the pyrene-labeled polymer and to determine the number (*N*_blob_) of SU that are encompassed inside a *blob*. Multiplying *N*_blob_ by the length (*b*) of a SU yields the contour length (*L*_c_) of the polymer segment inside a *blob* whose squared end-to-end distance (<*r*_EE_^2^>_blob_) represents the square of the *blob* diameter (see Figure 1). Conducting these PEF experiments with a series of poly(oligo(ethylene glycol) methyl ether methacrylate)s labeled with 1-pyrenebutanol (PyC_4_-PEG_n_MA) [19], *N*_blob_ was found to decrease with an increasing side chain length (*n*), reflecting the extension of the main chain. A plateau was reached for *n* equal to 16 and 19, corresponding to a fully extended polymethacrylate backbone over a length scale defined by a *blob*. Since a *blob* remains the same for a given polymer family, <*r*_EE_^2^>_blob_ could be determined by taking *N*_blob_ in the plateau region as *N*_blob_^∞^, corresponding to the *N*_blob_ value of a fully extended polymethacrylate backbone yielding <*r*_EE_^2^>_blob_ = (*N*_blob_^∞^ × *b*)^2^. Unfortunately, <*r*_EE_^2^>_blob_ was also found to decrease with increasing solvent viscosity as a consequence of PEF being diffusion-controlled, with a higher solvent viscosity reducing the reach of a pyrenyl label and <*r*_EE_^2^>_blob_. Fortunately, this detrimental effect could be eliminated by selecting organic solvents with a viscosity of 0.74 (±0.08) mPa.s at 25 °C, for which *N*_blob_^∞^ would equal 12 [19]. 

Because the *blobs* are much smaller than the polymer coil, *N*_blob_ takes values that are usually smaller than 100, which are small enough to ensure that *N*_blob_ is not affected by the solvent quality toward the polymer. Such conditions enable the application of the Kratky–Porod equation given in Equation (1) after being modified to reflect the confinement of a polymer segment inside a *blob* (see Figure 1) [23]. With *N*_blob_ known experimentally from the PEF measurements and *N*_blob_^∞^ and *b* being equal to, respectively, 12 [19] and 0.25 nm [24,25] for a methacrylate monomer, Equation (1) could be solved to retrieve *l*_p_ for the PyC_4_-PEG_n_MA samples in *N*,*N*-dimethylformamide (DMF) with a suitable 0.79 mPa.s solvent viscosity. *l*_p_ was found to increase linearly from 0.43 nm for PEG_0_MA (i.e., poly(methyl methacrylate)) to 1.8 nm for PEG_5_MA with the square of the number of non-hydrogen atoms in the side chain (*N*_S_^2^) of a PEG_n_MA sample as predicted theoretically [26].

(1) $$<r_{EE}^{2}{>}_{blob}\;=\;{\left(N_{blob}^{\infty }\times b\right)}^{2}\;=\;2l_{p}(b\times N_{blob})\;-\;2l_{p}^{2}\left[1\;-\;\exp\left(-\frac{b\times N_{blob}}{l_{p}}\right)\right]$$

While the *l*_p_ values retrieved for the PEG_n_MA samples in DMF by applying this PEF-based methodology were encouraging, they were the first *l*_p_ values reported for PEG_n_MA samples and could not be compared to already published *l*_p_ values. To validate the PEF-based methodology, the present study describes its application to a series of poly(alkyl methacrylate)s randomly labeled with 1-pyrenebutanol (PyC_4_-PC_n_MA with *n* = 1, 4, 6, 8, 12, and 18) to determine *l*_p_ for the PC_n_MA samples and compare them with already published *l*_p_ values obtained by viscometry [14]. The PEF experiments were conducted in *o*-xylene, which could solubilize all the PyC_4_-PC_n_MA samples and had a suitable viscosity of 0.76 mPa.s to ensure that *N*_blob_^∞^ equaled 12 [19]. The *l*_p_ values determined by PEF and viscometry [14] for the PC_n_MA samples were in good agreement, supporting the notion that the PEF-based methodology can be employed for *l*_p_ determination. The *l*_p_ values obtained with the PyC_4_-PC_n_MA samples in o-xylene were also compared to the *l*_p_ values obtained for the PyC_4_-PEG_n_MA samples in *o*-xylene and DMF [19] as well as those obtained for a series of PEG_n_MA in DMF where the pyrenyl labels were connected to the polymethacrylate backbone via a penta(ethylene glycol) linker (PyEG_5_-PEG_n_MA) [20]. The good agreement observed between the *l*_p_ values obtained for these different polymethacrylate samples with different side chain compositions in DMF for the PyC_4_-PEG_n_MA and PyEG_5_-PEG_n_MA samples, in *o*-xylene for the PyC_4_-PEG_n_MA and PyC_4_-PC_n_MA samples, and polymethacrylate backbones labeled with different pyrene derivatives indicates that the PEF-based methodology for determining *l*_p_ is robust.

While the pyrene-labeling requirement for PEF experiments conducted on macromolecules is a disadvantage compared to techniques like viscometry [14], scattering [15,16], or GPC [17,18] that can characterize a macromolecule without chemical post-modification, the extreme sensitivity of fluorescence enables the study of pyrene-labeled macromolecules in the 0.1–50 mg/L range, 3 to 4 orders of magnitude lower than what is currently achievable by the more conventional techniques. Consequently, this study demonstrates that the PEF-based methodology is a robust technique for probing the conformation of macromolecules, and because it can operate with extremely dilute solutions, it can effectively complement the more traditional characterization techniques under conditions requiring improved sensitivity, such as those encountered for macromolecules located at interfaces.

## 2. Materials and Methods

Chemicals: The synthesis and characterization of the poly(alkyl methacrylate)s labeled with 1-pyrenebutanol (PyC_4_-PC_n_MA with *n* = 1, 4, 6, 8, 12, and 18) and the poly(oligo(ethylene glycol) methyl ether methacrylate) labeled with either 1-pyrenebutanol (PyC_4_-PEG_n_MA with *n* = 0–5, 9, 16, and 19) or 1-pyrenemethoxypenta(ethylene glycol) (PyEG_5_-PEG_n_MA with *n* = 0, 3–5, 7, 9, and 19) were presented in the references [19,20,27], respectively. Their chemical structure is shown in Table 1. *o*-Xylene was purchased from Sigma-Aldrich (St. Louis, MO, USA).

UV-Vis Absorption: Absorption spectra of solutions of the PyC_4_-PC_n_MA and PyC_4_-PEG_n_MA samples in *o*-xylene were acquired with a Cary 100 UV-Visible spectrophotometer to ensure that the absorbance at 344 nm was equal to 0.1, corresponding to a concentration of pyrenyl labels of 2.5 × 10^−6^ M and a less than 50 mg/L concentration of pyrene-labeled polymer. Such low polymer concentrations ensure that no intermolecular PEF takes place.

Steady-State Fluorescence: All PyC_4_-PC_n_MA and PyC_4_-PEG_n_MA solutions in *o*-xylene were degassed with a gentle flow of nitrogen for 50 min before acquiring the fluorescence spectra and decays. A HORIBA QM-400 spectrofluorometer fitted with a xenon arc lamp was employed to acquire the fluorescence spectra of the PyC_4_-PC_n_MA and PyC_4_-PEG_n_MA solutions from 350 to 600 nm. The solutions were excited at 344 nm. Analysis of the fluorescence spectra yielded the *I*_E_/*I*_M_ ratio obtained by integrating the fluorescence spectra from 376 to 382 nm and from 500 to 530 nm to determine the monomer (*I*_M_) and excimer (*I*_E_) fluorescence intensity, respectively, before dividing *I*_E_ by *I*_M_.

Time-Resolved Fluorescence: The degassed solutions of PyC_4_-PC_n_MA and PyC_4_-PEG_n_MA in *o*-xylene were excited at 344 nm with a 340 nm NanoLED fitted to the excitation monochromator set at 344 nm of an IBH Ltd. time-resolved fluorometer with a 500 kHz repetition rate and a 2.04 ns time-per-channel to obtain their fluorescence decays at 379 and 510 nm for the pyrene monomer and excimer, respectively. The monomer and excimer fluorescence decays were acquired with cut-off filters at 370 and 495 nm placed before the emission monochromator to minimize stray light from reaching the detector and with 40,000 and 20,000 counts at the decay maximum, respectively. The beam from the NanoLED was reflected off a triangular aluminum monolith and passed through the emission monochromator set at 344 nm to obtain the instrument response function (IRF). 

Fluorescence Decay Analysis: The fluorescence *blob* model (FBM) was applied to fit globally the fluorescence decays of the pyrene monomer and excimer of the PyC_4_-PC_n_MA and PyC_4_-PEG_n_MA solutions in *o*-xylene [19,20]. The FBM acknowledges that, while it remains excited, a pyrenyl label only probes a finite subvolume of the polymer coil, referred to as a *blob*, which is used to compartmentalize the polymer coil into a cluster of identical *blobs* where the pyrenyl moieties distribute themselves randomly according to a Poisson distribution. The main parameters retrieved from the FBM analysis of the decays are the rate constant (*k*_blob_) for diffusive encounters taking place inside a *blob* between two structural units (SU) bearing an excited (*Py*_diff_*) and a ground-state pyrenyl labels, the average number <*n*> of ground-state pyrenyl groups inside a *blob*, and the product *k*_e_ × [*blob*] of the rate constant (*k*_e_) representing the exchange of pyrene groups between *blobs* and the *blob* concentration ([*blob*]) inside the polymer coil. Upon encounter between two SU bearing excited and ground-state pyrenes, the excited pyrene *Py*_diff_* turns into the species *Py*_k2_*, which reacts rapidly with the ground-state pyrene with the large rate constant *k*_2_ (~10 × *k*_blob_) to form one of the two excimers *E*0* or D*. *E*0* and D* represent pyrenyl labels that form an excimer with a well- or poorly stacked ground-state pyrene, resulting in a short or long excimer lifetime *τ*_E0_ or *τ*_D_, respectively. The fifth pyrene species accounts for the pyrenyl pendants that are isolated along the polymer, which cannot form excimer and emit as if they were free in solution (*Py*_free_*). The molar fractions *f*_diff_, *f*_k2_, and *f*_free_ represent the contributions from the species *Py*_diff_*, *Py*_k2_*, and *Py*_free_*, respectively, while the combined contribution of the pre-aggregated pyrene species *E*0* and D* are represented by the molar fraction *f*_agg_.

The mathematical expressions used to fit the fluorescence decays of the pyrene monomer and excimer are presented as Equations (S1) and (S2) in Supplementary Materials, respectively, after they had been convoluted with the IRF. The parameters used in Equations (S1) and (S2) were optimized according to the Marquardt-Levenberg algorithm [28] and these parameters retrieved from the global FBM analysis of the fluorescence decays are listed in Tables S1–S4. These FBM analyses were first conducted with the program *globmis90gbg* using a floating *k*_2_. The *k*_2_ values obtained by globally fitting the pyrene monomer and excimer fluorescence decays of all the PyC_4_-PC_n_MA or PyC_4_-PEG_n_MA samples for an *n*-value of a given polymer series were averaged. The analysis of these fluorescence decays was then repeated with the program *globmis90bbg* where *k*_2_ was fixed to its average value. This procedure has been shown to retrieve the other parameters with much greater accuracy [29]. A listing of the programs *globmis90gbg* and *globmis90bbg* written in Fortran is provided as Supplementary Materials.

The size of a *blob* could be determined from the number (*N*_blob_) of SU constituting a *blob* according to Equation (2). Equation (2) combines the molar fraction of SU bearing a pyrenyl label in the PyC_4_-PC_n_MA and PyC_4_-PEG_n_MA samples, which had been determined earlier [19,27], the molar fraction (*f*_Mfree_) of *Py*_free_* species detected in the pyrene monomer fluorescence decays, and the average number (<*n*>) of pyrenyl labels per *blob*.

(2) $$N_{blob}=(1-f_{Mfree})\times \frac{<n>}{x}$$

## 3. Results

As described in the Introduction, the application of PEF to retrieve the persistence length (*l*_p_) for the linear polymers PyC_4_-PEG_n_MA and PyEG_5_-PEG_n_MA requires a solvent with a viscosity equal to 0.74 (±0.08) mPa.s at 25 °C [19]. While the PEG_n_MA samples are soluble in many organic solvents, which enabled the identification of DMF with a 0.79 mPa.s viscosity as a suitable solvent for determining *l*_p_ by PEF measurements, dissolving a series of PC_n_MA samples with different alkyl chain lengths (*n*) in a same solvent is challenging. The difference in solubility between PC_n_MA and PEG_n_MA samples arises from the difference in polarity between the relatively polar polymethacrylate backbone and the pronounced decrease in the polarity of the samples experienced upon increasing the length (*n*) of the alkyl side chain for the PC_n_MA samples [13]. Many polar solvents like DMF, where PC_n_MA samples with small *n* values like poly(methyl methacrylate) (PC_1_MA) are soluble, cannot solubilize PC_n_MA samples with large *n* values such as poly(stearyl methacrylate) (PC_18_MA). The reverse holds true for apolar solvents like hexane, where PC_18_MA is soluble but PC_1_MA is not. Fortunately, all PC_n_MA samples are soluble in aromatic solvents like toluene (*η* = 0.56 mPa.s at 25 °C [19]) or *o*-xylene (*η* = 0.76 mPa.s at 25 °C [30]). Since the viscosity of *o*-xylene falls within the 0.74 (±0.08) mPa.s range required to retrieve *l*_p_ by PEF, the PyC_4_-PC_n_MA and PyC_4_-PEG_n_MA samples were studied in *o*-xylene to characterize their fluorescence properties and determine their persistence length.

### 3.1. Analysis of the Fluorescence Spectra

**Figure 2:**
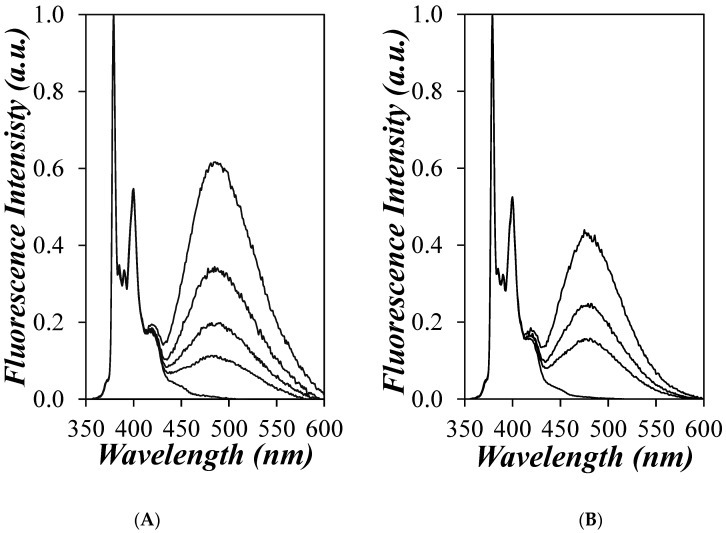
Fluorescence spectra of (**A**) the PyC_4_-PC_4_MA samples (from bottom to top: *x* = 0.003, 0.022, 0.030, 0.053, and 0.072) and (**B**) PyC_4_-PEG_1_MA samples (from bottom to top: *x* = 0.001, 0.038, 0.053, 0.075) in *o*-xylene. [*Py*] = 2.5 × 10^−6^ M, *λ*_ex_ = 344 nm.

The fluorescence spectra of the PyC_4_-PC_n_MA and PyC_4_-PEG_n_MA samples in *o*-xylene were acquired. Typical fluorescence spectra obtained for the PyC_4_-PC_4_MA and PyC_4_-PEG_1_MA series in *o*-xylene are presented in Figure 2A and Figure 2B, respectively, after being normalized at the 379 nm peak corresponding to the 0-0 transition of pyrene. The fluorescence spectra showed the spectral features expected for pyrene-labeled macromolecules, with the pyrene monomer exhibiting sharp peaks between 370 and 410 nm and the excimer emitting a broad structureless fluorescence centered at 480 nm. The fluorescence intensity of the pyrene excimer was found to increase relative to the fluorescence intensity of the pyrene monomer with increasing pyrene content as a result of increased pyrene-pyrene encounters. Similar figures were obtained for the fluorescence spectra of all the PyC_4_-PC_n_MA and PyC_4_-PEG_n_MA samples in *o*-xylene.

**Figure 3:**
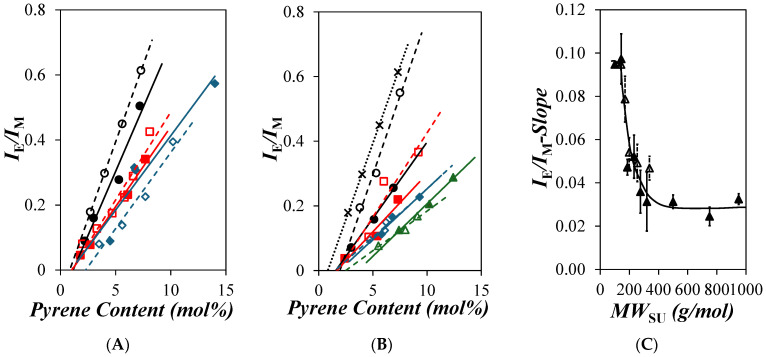
Plots of *I*_E_/*I*_M_ as a function of pyrene content for (**A**) the PyC4-PCnMA for *n* = (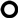) 1, (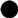) 4, (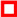) 6, (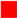) 8, (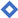) 12, and (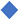) 18 and (**B**) the PyC4-PEGnMA samples for *n* = (×) 0, (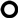) 1, (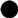) 2, (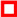) 3, (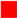) 4, (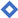) 5, (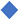) 9, (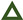) 16, and (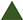) 19 in *o*-xylene and (**C**) *E*_PEF_ as a function of the molecular weight of a structural unit (*MW*_SU_) for the (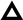) PyC_4_-PC_n_MA and (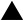) PyC_4_-PEG_n_MA samples.

The *I*_E_/*I*_M_ ratios were determined from the analysis of the fluorescence spectra, and they were plotted as a function of pyrene content in Figure 3A and Figure 3B for the PyC_4_-PC_n_MA and PyC_4_-PEG_n_MA samples, respectively. After the pyrene content reached an onset value, corresponding to the point where all the *blobs* along the polymethacrylate backbone contained at least one pyrenyl label, *I*_E_/*I*_M_ increased with increasing pyrene content, reflecting a higher encounter frequency between the pyrenyl labels. Approximating the increase in *I*_E_/*I*_M_ with increasing pyrene content as a straight line, the slope of those trends could be determined and was taken as the PEF efficiency (*E*_PEF_), which was plotted as a function of the molecular weight of a structural unit (*MW*_SU_) in Figure 3C for both series of PyC_4_-PC_n_MA and PyC_4_-PEG_n_MA samples in *o*-xylene. Within experimental error, the same *E*_PEF_-vs.-*MW*_SU_ trends were obtained for the PyC_4_-PC_n_MA and PyC_4_-PEG_n_MA series in *o*-xylene, reflecting the similar behavior of the two polymeric constructs despite the different polarity between the apolar alkyl and polar oligo(ethylene glycol) side chains.

*E*_PEF_ decreased with increasing *MW*_SU_ in Figure 3C until it reached a plateau for the PyC_4_-PEG_n_MA samples with a longer side chain. This behavior reflects the extension of the polymethacrylate backbone induced by the stronger steric hindrance generated by the longer side chains. As the length of the side chains increased further, their effect on the polymethacrylate backbone decreased until a further increase in side chain length no longer affected the conformation of the backbone, at which point *E*_PEF_ remained constant. The trend described for *E*_PEF_ as a function of *MW*_SU_ in Figure 3C indicates that PEF between the 1-pyrenebutyl groups bound to the polymethacrylate backbone reflects the backbone extension experienced by the PyC_4_-PC_n_MA and PyC_4_-PEG_n_MA samples as their side chain length is increased. Since the PEF signal displayed by these pyrene-labeled polymers reflects their conformation in solution, it must contain information about their persistence length. Indeed, the persistence length of these polymers can be determined through the analysis of their fluorescence decays, as described in the following section.

### 3.2. Fluorescence Decay Analysis

**Figure 4:**
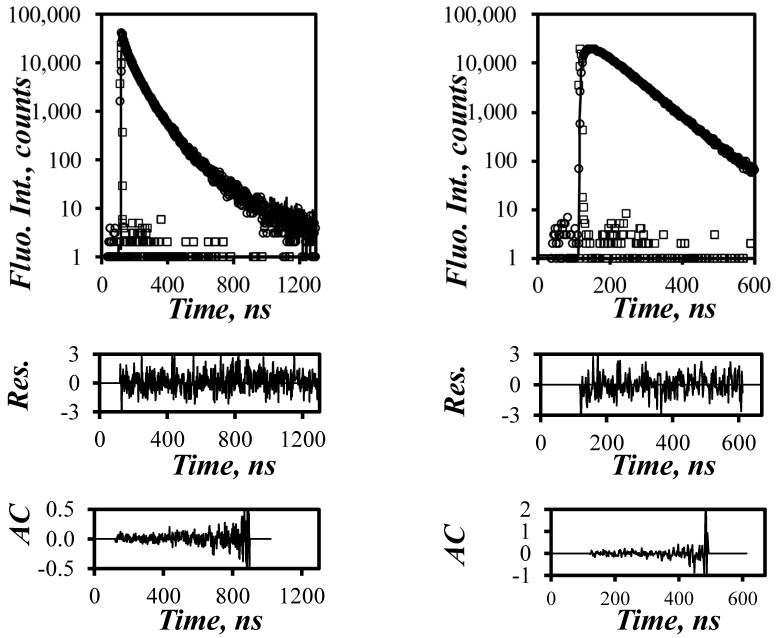
Fluorescence decays of (left, *l*_em_ = 379 nm) the pyrene monomer and (right, *l*_em_ = 510 nm) the pyrene excimer for the PyC_4_-PC_4_MA sample labeled with 7.2 mol% of pyrene. *L*_ex_ = 344 nm, [*Py*] = 2.5 × 10^−6^ M, *χ*^2^ = 1.20. In top panels: square: IRF, circles: experimental fluorescence decay, solid line: decay fit.

The fluorescence decays of the pyrene monomer and excimer were acquired for all the PyC_4_-PC_n_MA and PyC_4_-PEG_n_MA samples in *o*-xylene before being fitted globally according to the FBM with Equations (S1) and (S2) provided as Supplementary Materials. The quality of the fit was assessed from a *χ*^2^ value of less than 1.3 and the visual inspection of the random distribution around zero of the residuals and the autocorrelation of the residuals are illustrated in Figure 4. The parameters retrieved from these analyses are presented in Tables S1 and S2 as Supplementary Materials.

**Figure 5:**
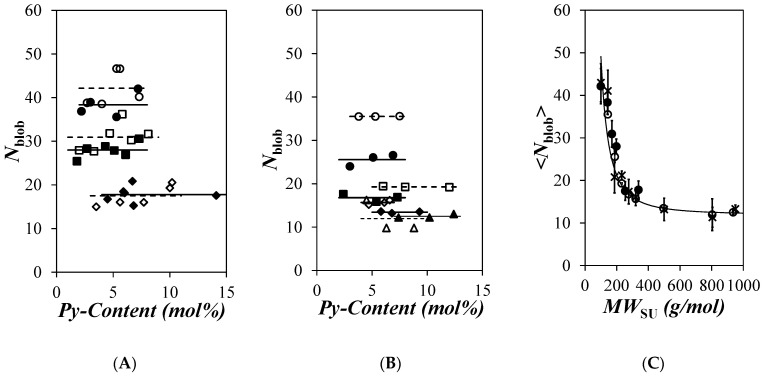
Plot of *N*_blob_ as a function of pyrene content for the (**A**) PyC4-PC_n_MA for *n* = (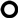) 1, (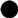) 4, (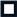) 6, (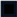) 8, (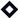) 12, and (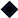) 18 and (**B**) PyC4-PEG_n_MA for *n* = (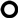) 1, (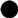) 2, (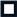) 3, (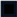) 4, (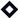) 5, (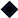) 9, (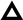) 16, and (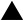) 19 in *o*-xylene and (**C**) comparison of the averaged <*N*_blob_> value of (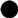) PyC4-PC_n_MA in *o*-xylene and PyC4-PEG_n_MA in (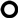) *o*-xylene and (×) DMF as a function of the molecular weight of a structural unit (*MW*_SU_). The solid line represents the predicted *N*_blob_-vs.-*MW*_SU_ trend for a 0.76 mPa.s solvent viscosity [19].

Among these parameters were the average number <*n*> of pyrenyl labels per *blob* and the molar fraction (*f*_Mfree_) of pyrenyl labels unable to form an excimer. These parameters were detected in the pyrene monomer fluorescence decay; they were combined in Equation (2) with the molar fraction (*x*) of pyrenyl labels attached to the polymers to yield the number (*N*_blob_) of SU inside a *blob*. *N*_blob_ was plotted as a function of the pyrene content for the different series of PyC_4_-PC_n_MA and PyC_4_-PEG_n_MA samples in Figure 5A and Figure 5B, respectively. Within experimental error, *N*_blob_ remained constant with pyrene content for all polymer samples, indicating that covalent attachment of the pyrene labels did not affect the behavior of the polymers. Averaging the *N*_blob_ values over all pyrene contents obtained for the same series of PyC_4_-PC_n_MA or PyC_4_-PEG_n_MA samples in *o*-xylene yielded <*N*_blob_>, which was plotted as a function of the molecular weight of a structural unit (*MW*_SU_) in Figure 5C. The trend obtained earlier for the PyC_4_-PEG_n_MA samples in DMF was added to Figure 5C [19]. 

As was previously observed for the PyC_4_-PC_n_MA samples in toluene and THF and for the PyC_4_-PEG_n_MA samples in a variety of solvents [19], <*N*_blob_> for the PyC_4_-PC_n_MA samples in *o*-xylene was found to decrease with increasing side chain length in Figure 5C reflecting the increased extension of the polymethacrylate backbone induced by the steric hindrance generated by the alkyl side chains. Within experimental error, the trends obtained for the PyC_4_-PC_n_MA and PyC_4_-PEG_n_MA samples in *o*-xylene presented in Figure 5C overlapped, indicating that both polymer families shared a similar conformation in *o*-xylene that extended with increasing side chain length. But although the preparation of the PyC_4_-PC_n_MA was limited to an *n* value of 18, the side chains of the PyC_4_-PEG_n_MA samples could be extended up to an *n* value of 19 representing a side chain with a number (*N*_S_) of non-hydrogen atom equal to 60 after including the atoms of the ester bond. As had been observed in other solvents, <*N*_blob_> for the PyC_4_-PEG_n_MA samples with *n* = 16 and 19 did not change, indicating that the backbone was fully extended. Furthermore, *N*_blob_^∞^ in Figure 3C obtained for the PyC_4_-PEG_n_MA samples with *n* = 16 and 19 equaled 12 (±2), as would be expected for an extended polymethacrylate backbone in a solvent with a viscosity of 0.74 (±0.08) mPa.s such as for *o*-xylene (*η* = 0.76 mPa.s) [19].

An earlier study established that the <*N*_blob_>-vs.-*MW*_SU_ trends obtained for the PyC_4_-PEG_n_MA samples in six different solvents was well-represented by a master equation depending solely on the solvent viscosity and *MW*_SU_ [19]. This master equation resulted in the solid line drawn in Figure 5C, which represents the predicted *N*_blob_-vs.-*MW*_SU_ trend for the PyC_4_-PEG_n_MA samples in *o*-xylene. As expected, the experimental <*N*_blob_> values for the PyC_4_-PEG_n_MA samples clustered around the predicted trend in Figure 5C. More surprisingly, considering the difference in polarity between the apolar alkyl side chains of the PyC_4_-PC_n_MA samples compared to the polar side chains of the PyC_4_-PEG_n_MA samples, the <*N*_blob_> values obtained for both polymer series in *o*-xylene clustered around the master curve. Similarly, the <*N*_blob_> values reported earlier for PyC_4_-PEG_n_MA in DMF, whose 0.79 mPa.s solvent viscosity was close to that of 0.76 mPa.s for *o*-xylene, also clustered around the *N*_blob_-vs.-*MW*_SU_ master trend in Figure 5C. The similar <*N*_blob_>-vs.-*MW*_SU_ trends shown in Figure 5C for the PyC_4_-PC_n_MA samples in *o*-xylene and for the PyC_4_-PEG_n_MA samples in *o*-xylene and DMF suggest that these polymers share the same polymethacrylate backbone and linear side chains adopt a same conformation that depends solely on the side chain length through *MW*_SU_. 

**Figure 6:**
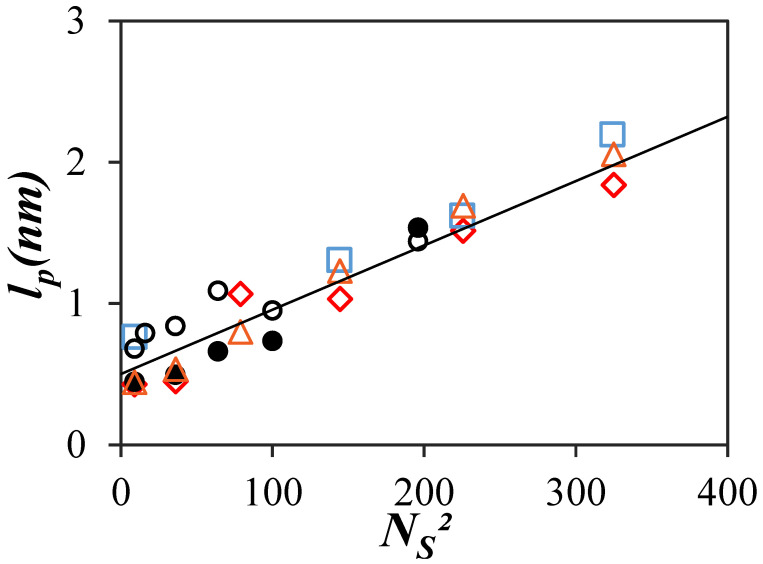
Plot of the persistence length (*l*_p_) as a function of the squared number of non-hydrogen atoms in the side chain (**N**_S_^2^) determined by (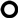) viscometry for PC_n_MA [14] and PEF for (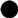) PyC_4_-PC_n_MA in *o*-xylene, (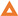) PyC_4_-PEG_n_MA in *o*-xylene, (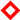) PyC_4_-PEG_n_MA in DMF [19], and (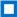) PyEG_5_-PEG_n_MA in DMF [20]. The solid line represents the predicted trend for *l*_p_ obtained with the PyC_4_-PEG_n_MA samples as a function of *N*_S_^2^ [19].

Equation (1) was then applied to extract the persistence length (*l*_p_) from the <*N*_blob_> values presented in Figure 5C for the PyC_4_-PC_n_MA and PyC_4_-PEG_n_MA samples in *o*-xylene using *b* and *N*_blob_^∞^ equal to 0.25 nm [14] and 12 [19], respectively. The *l*_p_ values obtained for the PyC_4_-PC_n_MA and PyC_4_-PEG_n_MA samples in *o*-xylene, the PyC_4_-PEG_n_MA [19], and PyEG_5_-PEG_n_MA [20] samples in DMF and those obtained by viscometry for a series of PC_n_Ma samples [14] were plotted as a function of the squared number of non-hydrogen atoms in the side chains (*N*_S_^2^). The solid line in Figure 6 is predicted after solving Equation (1) for *N*_blob_ values obtained from PyC_4_-PEG_n_MA samples dissolved in a hypothetical solvent with a viscosity of 0.74 mPa.s [19]. While the data presented in Figure 6 exhibit some scatter, they all clustered around the solid line, indicating a linear increase in *l*_p_ as a function of *N*_S_^2^ in agreement with theoretical predictions [26]. The overall agreement between the *l*_p_ values retrieved with different techniques (viscometry and PEF) for different families of polymers (poly(alkyl methacrylate)s and poly(oligo(ethylene glycol) methyl ether methacrylate)s) probed with different pyrene derivatives (1-pyrenebutanol and 1-pyrenemethoxy-penta(ethylene glycol)), and in different solvents (DMF and *o*-xylene) with a viscosity approaching that of 0.74 (±0.08) mPa.s suggest that the PEF-based methodology introduced earlier is robust and can be applied to determine *l*_p_ for polymers.

## 4. Discussion

The recent demonstration that PEF can probe the local density, and thus the conformation of macromolecules in solution [31], was taken advantage of in the present study to characterize the persistence length of a series of PyC_4_-PC_n_MA samples. The good agreement observed between the persistence lengths determined with different techniques and polymeric constructs in Figure 6 suggests that the methodology is robust. Since the present study confirms that PEF provides quantitative information about macromolecular conformations, the strengths and limitations of the PEF-based methodology are now discussed in comparison with the more traditional techniques already available.

Certainly, the main disadvantage of the PEF-based methodology described herein is the requirement that the macromolecule of interest is randomly labeled with pyrene, which imposes an additional synthetic step compared to techniques such as viscometry [14] or scattering [15,16], which use the macromolecule “as prepared”. Fortunately, the labeling must be random, which is much easier to accomplish compared to the specific end-labeling, which is typically required for quantitative studies of linear chains [32,33,34,35,36]. Random pyrene labeling can be achieved via grafting through*,* as is performed in the present and earlier studies [19,20,27,37] or grafting to as with polypeptides [38,39] or polysaccharides [40,41]. While random pyrene labeling can be fairly easily implemented, this extra synthetic step would not be needed with scattering or viscometry experiments.

Another limitation in the PEF-based methodology is the range of *l*_p_ values that can be retrieved. Since *l*_p_ is derived by using *N*_blob_ in Equation (1), *l*_p_ becomes more challenging to determine when *N*_blob_ approaches *N*_blob_^∞^, at which point *l*_p_ tends to infinity. As shown in Figure 5C, <*N*_blob_> ranges from 42 (±4) for PyC_4_-PC_1_MA to 18 (±2) for PyC_4_-PC_18_MA, the latter value approaching the *N*_blob_^∞^ value of 12. In fact, a 1.65 nm *l*_p_ value was obtained for PyC_4_-PC_18_MA with *N*_S_^2^ = 400, which would have been off the straight line in Figure 6, suggesting that the <*N*_blob_> value of 18 for PC_4_-PC_18_MA might be too close to *N*_blob_^∞^ to recover an accurate *l*_p_. Larger *N*_blob_ values can be retrieved by using a pyrene derivative with a longer linker since a longer linker enables a pyrenyl label to probe a larger *blob* resulting in a larger *N*_blob_ value [37,39]. However, the linker should not be too long, as in the case of the penta(ethylene glycol) linker used for the PyEG_5_-PEG_n_MA samples, which could not fully deploy during the time a pyrenyl label remained excited despite the long lifetime of pyrene [20]. *N*_blob_^MMO^ obtained from molecular mechanics optimization (MMO) for the PyEG_5_-PEG_n_MA samples was found to equal 41, whereas <*N*_blob_> only equaled 23 (±2) for the “fully extended” PyEG_5_-PEG_19_MA sample [42], a clear indication that the pyrenyl label did not have sufficient time to fully deploy in solution. In summary, the PEF-based methodology is unlikely to yield the persistence length of stiff polymeric backbones like that of DNA or a-helical poly(*L*-glutamic acid). Nevertheless, the still unknown persistence length of numerous polymers with more flexible backbones could be determined with the PEF-based methodology described in this report.

Another complication associated with PEF-based experiments is that the macromolecule under study should be photochemically inert over the range of wavelengths involved for the PEF experiments, with the pyrene derivatives being typically excited at 344 nm and their fluorescence being monitored between 360 and 600 nm (see Figure 2). This condition precludes the application of PEF to fullerenes, carbon nanotubes, and many conjugated polymers whose spectral features interfere with those of pyrene.

Despite these drawbacks, the PEF-based methodology offers several advantages that should be considered over the more traditional techniques. The first one is that the use of a *blob* as a unit volume, which is much smaller than the polymer coil, makes the methodology impervious to the polydispersity of the polymer sample. Since a large or short polymer chain is described by many or few identical *blobs*, respectively, the focus of the study is shifted from the entire population of polydisperse chains to that of identical monodisperse *blobs*. This aspect represents a major advantage of the PEF-based methodology since it enables the study of polydisperse samples such as the PyC_4_-PC_n_MA and PyC_4_-PEG_n_MA samples investigated herein. Such polymer samples would otherwise be more challenging to characterize by scattering or viscosity experiments, which are much more sensitive to sample polydispersity.

The second advantage is the extreme sensitivity of PEF. Solutions with concentrations of pyrene-labeled polymers as low as 0.5 mg/L yield enough fluorescence signal to acquire the fluorescence spectra and decays required for a fluorescence analysis. While the use of traditional techniques such as scattering or viscometry always requires the extrapolation of trends obtained as a function of polymer concentration to zero-polymer concentration, this extrapolation step is irrelevant in fluorescence experiments since these experiments are conducted at polymer concentrations that are so low that they are equivalent to zero-polymer concentrations. This feature is most advantageous when working with macromolecules that are subject to intermacromolecular long-range electrostatic forces for protonated PAMAM dendrimers [43] or are poorly soluble and might aggregate by working at higher concentrations. Consequently, and like any other technique, the PEF-based methodology described in this report possesses disadvantages and advantages that need to be considered before deciding on its application for the study of a macromolecule.

## 5. Conclusions

Although the chemical composition of the PyC_4_-PC_n_MA and PyC_4_-PEG_n_MA samples is quite different, with the side chains of the latter and former polymers being polar oligo(ethylene glycol) and apolar alkyl chains, both polymers shared similar conformations in solvents where they were both soluble, such as in THF [19], toluene [19], and now *o*-xylene based on their similar <*N*_blob_>-vs.-*MW*_SU_ trends. Furthermore, the fact that the <*N*_blob_>-vs.-*MW*_SU_ trend obtained for PyC_4_-PEG_n_MA in DMF overlapped those of PyC_4_-PC_n_MA and PyC_4_-PEG_n_MA in *o*-xylene further supported the notion that these trends depended solely on the solvent viscosity (and not its nature) and *MW*_SU_, as shown in Figure 5C. This outcome resulted from the similar architecture displayed by these polymer samples, which shared a common polymethacrylate backbone and linear side chains.

The similar conformations expected from the <*N*_blob_>-vs.-*MW*_SU_ trends shown in Figure 5C for the PyC_4_-PC_n_MA and PyC_4_-PEG_n_MA samples in *o*-xylene were confirmed by applying Equation (1) to extract *l*_p_. *l*_p_ was found to increase linearly with increasing *N*_S_^2^ in Figure 6, as expected from theoretical predictions [26]. Within experimental error, all *l*_p_-vs.-*N*_S_^2^ trends shown in Figure 6 agreed with each other, confirming the ability of these PEF experiments to retrieve *l*_p_. Furthermore, the *l*_p_ values obtained from the PEF experiments with the PyC_4_-PC_n_MA samples in *o*-xylene were in good agreement with those reported for PC_n_MA samples obtained by viscometry, which validated the *l*_p_ values retrieved from PEF measurements. Consequently, this study confirms the capability of PEF experiments to characterize the conformation of linear chains in solution through the determination of *l*_p_. By taking advantage of fluorescence to conduct these experiments at very low polymer concentrations, the PEF-based methodology described herein complements the more traditional techniques, such as viscometry, scattering, and GPC, which are also used to determine *l*_p_ but at usually much higher polymer concentrations.

## Figures and Tables

**Table 1:** Chemical structure of the polymer samples PyC_4_-PC_n_MA, PyC_4_-PEG_n_MA, and PyEG_5_-PEG_n_MA.

**PyC_4_-PC_n_MA**	**PyC_4_-PEG_n_MA**	**PyEG_5_-PEG_n_MA**
n = 1, 4, 6, 8, 12, and 18	n = 0–5, 9, 16, and 19	n = 0, 3–5, 7, 9, and 19
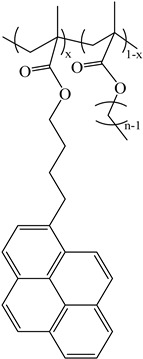	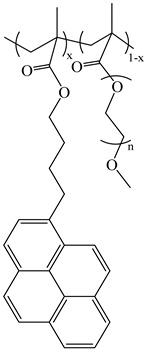	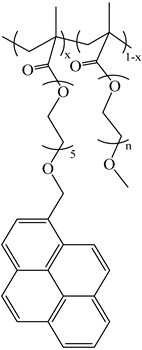

## Data Availability

Data are contained within the article and Supplementary Materials.

## Supplementary Materials

The following supporting information can be downloaded at https://www.mdpi.com/article/10.3390/polym16152126/s1, Equations used for the fluorescence blob model (FBM) analysis of the fluorescence decays, lists of parameters retrieved from the FBM analysis of the fluorescence decays, and listings of the programs *globmis90gbg* and *globmis90bbg*.
