# Supplementary material for: Flexibility of Poly(alkyl methacrylate)s Characterized by Their Persistence Length Determined through Pyrene Excimer Formation
Source: Polymers (Basel). 2024 Jul 26;16(15):2126. doi: 10.3390/polym16152126 (PMC11314411; doi:10.3390/polym16152126)
Supplement: Supplementary file 1 [file polymers-16-02126-s001.zip › polymers-3118781-supplementary.pdf]

Supporting Material

for

Flexibility of Poly(Alkyl Methacrylate)s Characterized from their Persistence Length Determined by Pyrene Excimer Formation

Kristijan Lulic, Grégoire Muller, Renzo Gutierrez, Hunter Little, Jean Duhamel*

Institute for Polymer Research, Waterloo Institute for Nanotechnology, Department of Chemistry, University of Waterloo, 200 University Avenue West, Waterloo, ON N2L 3G1, Canada

* To Whom correspondence should be addressed.

e-mail addresses: klulic@uwaterloo.ca; gmuller004@enscbp.fr; renzo.gutierrez@uwaterloo.ca; htlittle@uwaterloo.ca; jduhamel@uwaterloo.ca

Table of Contents

A] Equations used for the FBM analysis of the fluorescence decays 2-3

B] Lists of parameters retrieved form the FBM analysis of the fluorescence decays 4-7

C] Listing of the program *globmis90gbg* 8-49

D] Listing of the program *globmis90bbg* 50-89

A] Equations used for the FBM

Equation S1 and S2 were used to fit the monomer and excimer decays globally according to the FBM.

$$[Py^*] = \left[Py_{diff}{}^*\right]_{t=0} \exp\left(-\left(A_2 + \frac{1}{\tau_M}\right)t - A_3\left(1-\exp(-A_4 t)\right)\right) +$$
$$\left([Py_{diff}{}^*]_{t=0} + \left[Py_{diff}{}^*\right]_{t=0} \exp\left(A_3\right)\sum_{i=0}^{\infty}\frac{A_3^{\,i}}{i!}\frac{A_2 + iA_4}{A_2 + iA_4 - k_2}\right)\exp\left(-\left(k_2 + \frac{1}{\tau_M}\right)t\right)$$
$$-\left[Py_{diff}{}^*\right]_{t=0} \exp\left(A_3\right)\sum_{i=0}^{\infty}\frac{A_3^{\,i}}{i!}\frac{A_2 + iA_4}{A_2 + iA_4 - k_2}\exp\left(-\left(A_2 + iA_4 + \frac{1}{\tau_M}\right)t\right)$$
$$+[Py_{free}{}^*]_{t-0}\exp\left(-\frac{t}{\tau_M}\right) \tag{S1}$$

$$[E^*] = k_2\left([Py_{k2}{}^*]_{t=0} + [Py_{diff}{}^*]_{t=0}\exp(-A_3)\sum_{i=0}^{\infty}\frac{A_3^{\,i}}{i!}\frac{A_2 + iA_4}{A_2 + iA_4 - k_2}\right)$$
$$\times\frac{\exp\left(-\frac{t}{\tau_{E0}}\right) - \exp\left(-\left(k_2 + \frac{1}{\tau_M}\right)t\right)}{k_2 + \frac{1}{\tau_M} - \frac{t}{\tau_{E0}}} + [Py_{diff}{}^*]_{t=0}\exp(-A_3)$$
$$\times\sum_{i=0}^{\infty}\frac{A_3^{\,i}}{i!}\frac{A_2 + iA_4}{A_2 + iA_4 - k_2}\frac{\exp\left(-\left(A_2 + iA_4 + \frac{1}{\tau_M}\right)t\right) - \exp\left(-\frac{t}{\tau_{E0}}\right)}{A_2 + iA_4 + \frac{1}{\tau_M} - \frac{1}{\tau_{E0}}}$$
$$+[E0^*]_{t=0}\times\exp\left(-\frac{t}{\tau_{E0}}\right) + [EL^*]_{t=0}\times\exp\left(-\frac{t}{\tau_{EL}}\right) \tag{S2}$$

Equations S1 and S2 use the parameters A_2, A_3, and A_4, which are described in Equations S3.

$$A_2 = \langle n \rangle \frac{k_{blob}k_e[blob]}{k_{blob} + k_e[blob]} \tag{S3a}$$

$$A_3 = \langle n \rangle \left(\frac{k_{blob}}{k_{blob} + k_e[blob]}\right)^2 \tag{S3b}$$

$$A_4 = k_{blob} + k_e[blob] \tag{S3c}$$

In Equations S3, $<n>$ is the average number of ground-state pyrenyl labels per *blob*, k_{blob} is the rate constant for encounters between two structural units bearing a pyrenyl label, and $k_e \times [blob]$ describes the exchange of ground-state pyrenyl labels among *blobs*.

B] Lists of parameters retrieved from the FBM of the fluorescence decays

Table S1. Parameters retrieved from the monomer of the PyC_4-PEG_nMA series in *o*-xylene fit with the program *globmis90bbg* where k_2 is fixed in the analysis.

Sample	Mol %	k_{blob} (10^{-6} s^{-1})	$<n>$	k_e[blob] (10^{-6} s^{-1})	f_{Mdiff}	f_{k2}	f_{Mfree}	χ^2
PyC_4-PEG_1MA $k_2 = 1.30 \times 10^{-8}$ s^{-1} $\tau_M = 179$ ns	3.8	6.08	1.45	6.46	0.77	0.16	0.07	1.15
	5.3	7.30	1.98	7.30	0.72	0.23	0.05	1.10
	7.5	7.51	2.75	8.64	0.67	0.30	0.03	1.08
PyC_4-PEG_2MA $k_2 = 0.89 \times 10^{-8}$ s^{-1} $\tau_M = 174$ ns	3.0	4.98	1.00	6.47	0.61	0.11	0.28	1.12
	5.1	4.83	1.46	5.66	0.74	0.17	0.09	1.19
	6.9	5.22	1.93	6.11	0.72	0.23	0.05	1.02
PyC_4-PEG_3MA $k_2 = 1.19 \times 10^{-8}$ s^{-1} $\tau_M = 157$ ns	6.0	9.45	1.24	7.81	0.72	0.23	0.06	1.14
	8.0	7.71	1.73	7.71	0.66	0.24	0.11	1.05
	12.0	11.37	2.35	13.40	0.55	0.43	0.02	1.13
PyC_4-PEG_4MA $k_2 = 0.87 \times 10^{-8}$ s^{-1} $\tau_M = 167$ ns	2.4	9.65	0.62	7.38	0.55	0.13	0.32	1.08
	5.4	5.68	1.10	5.03	0.60	0.18	0.22	1.10
	7.3	7.24	1.40	8.74	0.63	0.25	0.12	1.12
PyC_4-PEG_5MA $k_2 = 0.93 \times 10^{-8}$ s^{-1} $\tau_M = 175$ ns	4.7	7.47	0.87	5.44	0.67	0.15	0.18	1.17
	6.1	6.86	1.18	5.37	0.61	0.19	0.19	1.05
	6.6	9.40	1.23	11.78	0.63	0.25	0.13	1.25
PyC_4-PEG_9MA $k_2 = 1.04 \times 10^{-8}$ s^{-1} $\tau_M = 181$ ns	5.8	8.38	0.95	7.24	0.65	0.18	0.17	1.01
	6.8	10.15	1.01	8.31	0.68	0.21	0.11	1.12
	9.3	8.84	1.34	6.50	0.68	0.26	0.06	1.04
PyC_4-$PEG_{16}MA$ $k_2 = 0.97 \times 10^{-8}$ s^{-1} $\tau_M = 167$ ns	4.5	11.15	0.95	6.87	0.55	0.23	0.23	1.02
	6.3	10.03	1.03	7.08	0.44	0.16	0.40	0.98
	8.8	9.02	1.20	5.51	0.49	0.24	0.28	0.98
PyC_4-$PEG_{19}MA$ $k_2 = 0.96 \times 10^{-8}$ s^{-1} $\tau_M = 172$ ns	7.4	8.37	1.13	4.44	0.61	0.19	0.20	1.14
	10.2	8.22	1.37	3.29	0.67	0.24	0.09	1.13
	12.4	7.69	1.72	4.11	0.63	0.31	0.06	1.13

Table S2. Parameters retrieved from the excimer of the PyC$_4$-PEG$_n$MA series in *o*-xylene fit with the program *globmis90bbg* where k_2 is fixed in the analysis.

Sample	Mol %	f_{Ek2}	τ_{E0} (ns)	f_{E0diff}	f_{EE0}	τ_{EL} (ns)	f_{EEL}	χ^2
PyC$_4$-PEG$_1$MA $k_2 = 1.30 \times 10^{-8}$ s^{-1}	3.8	0.17	53	0.80	0.0004	121	0.03	1.15
	5.3	0.23	52	0.71	0.0000	86	0.06	1.10
	7.5	0.28	50	0.62	0.0119	81	0.09	1.08
PyC$_4$-PEG$_2$MA $k_2 = 0.89 \times 10^{-8}$ s^{-1}	3.0	0.14	56	0.82	0.0000	151	0.04	1.12
	5.1	0.18	50	0.78	0.0014	122	0.05	1.19
	6.9	0.22	51	0.71	0.0423	119	0.03	1.02
PyC$_4$-PEG$_3$MA $k_2 = 1.19 \times 10^{-8}$ s^{-1}	6.0	0.23	56	0.72	0.0520	322	0.00	1.14
	8.0	0.25	51	0.69	0.0002	108	0.06	1.05
	12.0	0.39	50	0.50	0.0140	79	0.10	1.13
PyC$_4$-PEG$_4$MA $k_2 = 0.87 \times 10^{-8}$ s^{-1}	2.4	0.19	58	0.79	0.0000	182	0.02	1.08
	5.4	0.22	53	0.75	0.0001	152	0.03	1.10
	7.3	0.25	52	0.68	0.0330	145	0.02	1.12
PyC$_4$-PEG$_5$MA $k_2 = 0.93 \times 10^{-8}$ s^{-1}	4.7	0.18	52	0.77	0.0000	123	0.05	1.17
	6.1	0.23	50	0.72	0.0028	121	0.05	1.05
	6.6	0.27	51	0.69	0.0205	151	0.02	1.25
PyC$_4$-PEG$_9$MA $k_2 = 1.04 \times 10^{-8}$ s^{-1}	5.8	0.22	54	0.76	0.0193	263	0.00	1.01
	6.8	0.23	54	0.73	0.0480	804	0.00	1.12
	9.3	0.27	53	0.69	0.0377	144	0.01	1.04
PyC$_4$-PEG$_{16}$MA $k_2 = 0.97 \times 10^{-8}$ s^{-1}	4.5	0.26	56	0.63	0.0823	158	0.03	1.02
	6.3	0.26	52	0.70	0.0001	146	0.04	0.98
	8.8	0.31	53	0.64	0.0274	146	0.03	0.98
PyC$_4$-PEG$_{19}$MA $k_2 = 0.96 \times 10^{-8}$ s^{-1}	7.4	0.23	49	0.72	0.0001	118	0.04	1.14
	12.2	0.25	48	0.67	0.0007	96	0.08	1.13
	12.4	0.31	51	0.62	0.0521	136	0.02	1.13

Table S3. Parameters retrieved from the monomer of the PyC_4-PC_nMA series in *o*-xylene fit with the program *globmis90bbg* where k_2 is fixed in the analysis.

Sample	Mol %	k_{blob} (10^{-6} s^{-1})	<n>	k_e[blob] (10^{-6} s^{-1})	f_{Mdiff}	f_{k2}	f_{Mfree}	χ^2
PyC_4-PC_1MA $k_2 = 1.34 \times 10^{-8}$ s^{-1} τ_M = 176 ns	2.7	7.57	1.13	7.21	0.79	0.13	0.08	1.09
	4.0	7.78	1.63	9.58	0.77	0.18	0.06	1.09
	5.6	6.08	2.65	6.74	0.71	0.27	0.02	1.08
	7.3	7.33	2.99	8.13	0.67	0.31	0.02	1.20
PyC_4-PC_4MA $k_2 = 0.96 \times 10^{-8}$ s^{-1} τ_M = 186 ns	2.2	5.23	0.967	6.15	0.71	0.12	0.16	1.18
	3.0	5.57	1.28	4.86	0.74	0.17	0.09	1.15
	5.3	5.24	1.93	5.66	0.71	0.26	0.02	0.98
	7.2	5.08	3.07	4.22	0.61	0.37	0.01	1.14
PyC_4-PC_6MA $k_2 = 0.88 \times 10^{-8}$ s^{-1} τ_M = 176 ns	2.0	6.89	0.838	7.38	0.55	0.11	0.33	1.09
	3.3	5.72	1.08	6.04	0.68	0.16	0.15	1.09
	4.7	4.34	1.63	3.46	0.70	0.21	0.08	1.26
	5.8	3.54	2.21	1.97	0.68	0.26	0.05	1.29
	6.6	4.85	2.11		0.65	0.29	0.06	1.15
	8.1	4.64	2.61	3.88	0.63	0.35	0.02	1.10
PyC_4-PC_8MA $k_2 = 0.77 \times 10^{-8}$ s^{-1} τ_M = 182 ns	1.8	5.30	0.77	6.37	0.50	0.09	0.41	1.08
	2.7	4.25	0.81	4.52	0.81	0.13	0.06	1.16
	6.1	4.62	1.70	4.26	0.67	0.29	0.04	1.04
	7.7	4.44	2.27	3.50	0.65	0.33	0.02	1.11
PyC_4-$PC_{12}MA$ $k_2 = 0.83 \times 10^{-8}$ s^{-1} τ_M = 176 ns	3.5	7.16	0.86	7.09	0.48	0.13	0.39	0.99
	5.6	6.38	1.09	4.80	0.63	0.20	0.17	1.07
	7.7	6.64	1.32	4.67	0.66	0.27	0.07	1.09
	10.2	5.36	2.12	5.36	0.61	0.37	0.01	1.05
PyC_4-$PC_{18}MA$ $k_2 = 0.78 \times 10^{-8}$ s^{-1} τ_M = 179 ns	4.5	5.43	0.96	4.51	0.62	0.16	0.22	1.22
	6.7	8.26	1.58	5.04	0.60	0.29	0.11	1.06
	6.8	5.91	1.18	3.14	0.64	0.24	0.12	1.07
	14	5.69	2.58	4.53	0.47	0.49	0.04	1.08

Table S4. Parameters retrieved from the excimer of the Py-PAMA series in *o*-xylene fit with the program *globmis90bbg* where k_2 is fixed in the analysis.

Sample	Mol %	f_{Ek2}	τ_{E0} (ns)	f_{E0diff}	f_{EE0}	τ_{EL} (ns)	f_{EEL}	χ^2
PyC4-PC1MA $k_2 = 1.34 \times 10^{-8}$ s^{-1}	2.7	0.14	57	0.82	0.03	233	0.004	1.09
	4.0	0.18	52	0.77	0.03	132	0.02	1.09
	5.6	0.26	53	0.69	0.05	150	0.005	1.08
	7.3	0.29	52	0.63	0.06	109	0.02	1.20
PyC4-PC4MA $k_2 = 0.96 \times 10^{-8}$ s^{-1}	2.2	0.15	54	0.84	0.01	443	0.04	1.19
	3.0	0.18	53	0.78	0.01	129	0.02	1.11
	5.3	0.18	55	0.78	0.03	190	0.004	1.15
	7.2	0.34	50	0.57	0.02	85	0.07	1.14
PyC4-PC6MA $k_2 = 0.88 \times 10^{-8}$ s^{-1}	2.0	0.16	58	0.79	0.02	192	0.02	1.09
	3.3	0.18	55	0.77	0.04	201	0.005	1.09
	4.7	0.22	52	0.73	0.02	128	0.02	1.26
	5.8	0.27	52	0.70	0.01	143	0.01	1.29
	6.6							1.15
	8.1	0.33	52	0.59	0.07	134	0.01	1.10
PyC4-PC8MA $k_2 = 0.77 \times 10^{-8}$ s^{-1}	1.8	0.15	56	0.81	0.03	194	0.01	1.08
	2.7	0.12	45	0.80	0.0000	95	0.07	1.16
	6.1	0.28	54	0.66	0.06	199	0.001	1.04
	7.7	0.31	51	0.60	0.06	116	0.02	1.11
PyC4-PC12MA $k_2 = 0.83 \times 10^{-8}$ s^{-1}	3.5	0.21	54	0.75	0.009	172	0.03	0.99
	5.6	0.23	53	0.72	0.02	144	0.03	1.07
	7.7	0.27	53	0.65	0.04	122	0.03	1.09
	10.2	0.35	50	0.57	0.02	96	0.06	1.05
PyC4-PC18MA $k_2 = 0.78 \times 10^{-8}$ s^{-1}	4.5	0.20	52	0.76	0.04	350	0.0006	1.22
	6.7	0.30	50	0.62	0.0000	108	0.07	1.06
	6.8	0.26	50	0.68	0.007	122	0.05	1.07
	14	0.44	52	0.42	0.1	107	0.03	1.08

C] Listing of the program *globmis90gbg*:

```
c      Program created on October 11th, 2007.

c      This program fits the monomer and excimer decays simultaneously
c      with the blob model equation.

c      Excimer formation occur sequentially. Two monomers diffuse with
c      the blob model equation, form a pyrene aggregate that
rearranges
c      quickly with a rate constant k2 to form the excimer. k2 is
c      optimized in the analysis.

c      The excimer decay is fitted with the blob model equation + a
c      monoexponential function for excited ground-state dimers whose
c      pre-exponential factor and decaytime are fitted in the
analysis.

c      There is an extra lifetime for the excimer to account for
c      long-lived ground-state dimers.

       implicit double precision (a-h,o-z)

       parameter (mfit=16,ma=16,nca=16,nexp=1,niter=11)

       dimension y1(2000),lista(mfit),a(mfit),
     & covar(mfit,mfit),alpha(mfit,mfit),
     & e1(2000),da(mfit),beta(mfit)
     & ,dyda(mfit),ym(2000),res1(2000),auto1(2000)
     & ,e2(2000),y2(2000),res2(2000),auto2(2000)
     & ,tid1(nexp),tidi1(nexp),ted1(niter),tedi1(niter),
     & div0(niter),div1(nexp),div2(niter,nexp),aa0(niter)
     & ,aE(nexp),tauE(nexp),nstart1(3),nstart2(3)

       real*8 tauM,chisq,tpc1,tpc2


10     format(A)
       character *30 DD1,DD2


       write (*,*) 'How many chanels do you want to work with
     & for the monomer decay?'
       read (*,*) ndata1

c      write (*,*) 'What is the name of your lamp file ?'
c      read(*,10) DD
```

```
c       open (1,file=DD,status='old')

c       read(1,*) (e(i),i=1,ndata)

c       close(1)

c       write (*,*) 'what is the name of your fluorescence decay?'
c       read(*,10) DD

c       open(1,file=DD,status='old')

c       read(1,*) (y(i),i=1,ndata)

c       close(1)

c       write (*,*) 'What is your file''s name for the monomer?'
c       read(*,10) DD1

c       open(1,file=DD1,status='old')

c       do 15 i=1,ndata1

c       read(1,*) e1(i),y1(i)

c15     continue

c       close (1)

        write (*,*) 'What is your lamp''s filename for
     & the monomer decay?'
        read(*,10) DD1

        open(1,file=DD1,status='old')

        do 13 i=1,9
13      read(1,*)

        do 15 i=1,ndata1

        read(1,*) x,e1(i)

15      continue

        close (1)
```

```
      write (*,*) 'What is your monomer decay''s filename?'
      read(*,10) DD1

      open(1,file=DD1,status='old')

      do 14 i=1,9
14    read(1,*)

      do 16 i=1,ndata1

      read(1,*) x,y1(i)

16    continue

      close (1)


      write (*,*) 'How many chanels do you want to work with
     & for the excimer decay?'
      read (*,*) ndata2

c     write (*,*) 'What is your file''s name for the excimer?'
c     read(*,10) DD2

c     open(1,file=DD2,status='old')

c     do 16 i=1,ndata2

c     read(1,*) e2(i),y2(i)

c16   continue

c     close (1)


      write (*,*) 'What is your lamp''s filename for
     & the excimer decay?'
      read(*,10) DD2

      open(1,file=DD2,status='old')

      do 313 i=1,9
313   read(1,*)

      do 315 i=1,ndata2
```

```
        read(1,*) x,e2(i)

315     continue

        close (1)


        write (*,*) 'What is your excimer decay''s filename?'
        read(*,10) DD2

        open(1,file=DD2,status='old')

        do 314 i=1,9
314     read(1,*)

        do 316 i=1,ndata2

        read(1,*) x,y2(i)

316     continue

        close (1)


c       write (*,*) 'What is your lamp''s filename for the monomer?'
c       read(*,10) DD1

c       open(1,file=DD1,status='old')

c       read(1,*)

c       do 15 i=1,ndata1

c       read(1,*) e1(i)

c15     continue

c       close (1)

c       write (*,*) 'What is your decay''s filename for the monomer?'
c       read(*,10) DD1

c       open(1,file=DD1,status='old')

c       read(1,*)
```

```
c      do 16 i=1,ndata1

c      read(1,*) y1(i)

c16    continue

c      close (1)


c      write (*,*) 'What is your lamp''s filename for the excimer?'
c      read(*,10) DD2

c      open(1,file=DD2,status='old')

c      read(1,*)

c      do 315 i=1,ndata2

c      read(1,*) e2(i)

c315   continue

c      close (1)

c      write (*,*) 'What is your decay''s filename for the excimer?'
c      read(*,10) DD2

c      open(1,file=DD2,status='old')

c      read(1,*)

c      do 316 i=1,ndata2

c      read(1,*) y2(i)

c316   continue

c      close (1)

       write (*,*) 'What is the lifetime of the reference compound
     & for the monomer decays?'
       read(*,*) taur1

       write (*,*) 'What is your time per chanel for the monomer
decays?'
```

```
        read(*,*) tpc1

        taur1 = exp(-tpc1/taur1)

        alold2 = e1(1)

        do 40 i=2,ndata1

        alold1 = e1(i)
        e1(i) = e1(i) - alold2*taur1
        alold2 = alold1

        if(e1(i).lt.0) e1(i)=0.0001

40      continue


        write (*,*) 'What is the lifetime of the reference compound
      & for the excimer decays?'
        read(*,*) taur2

        write (*,*) 'What is your time per chanel for the excimer
decays?'
        read(*,*) tpc2

        taur2 = exp(-tpc2/taur2)

        alold2 = e2(1)

        do 41 i=2,ndata2

        alold1 = e2(i)
        e2(i) = e2(i) - alold2*taur2
        alold2 = alold1

        if(e2(i).lt.0) e2(i)=0.0001

41      continue

        imax = 0

        do 500 i=2,ndata1

        if (e1(i).gt.emax) then
        imax = i
        emax = e1(i)
```

```
      endif

500   continue


      write (*,511)
      write (*,*) '________________________________________________'
511   format(10H   channel,10H      lamp,10H     decay)

      do 520 k=imax-15,imax+10

      if (k.gt.1) then
      write (*,512) k,e1(k),y1(k)
512   format (3H   ,I4,3H   ,2(2H  ,F7.1,1H ))
      endif
520   continue

      write (*,*) 'From which channel do you wish to start
     & your analysis for the monomer decay?'
      read(*,*) nstart1(3)

      write (*,*) 'At which channel does the background noise begin
     & for the monomer decay?'
      read(*,*) nstart1(1)

      write (*,*) 'At which channel does the background noise end
     & for the monomer decay?'
      read(*,*) nstart1(2)

c     sume = 0.0
c     sumy = 0.0

c     anback = nback2-nback1+1

c     do 860 i=nback1,nback2

c     sume = sume + e1(i)
c     sumy = sumy + y1(i)

c860  continue

c     sume = sume/anback
c     sumy = sumy/anback

c     do 870 i=1,ndata1
```

```
c       e1(i) = e1(i) - sume
c       y1(i) = y1(i) - sumy

c       if(e1(i).lt.0.0) e1(i) = 0.00001
c       if(y1(i).lt.0.0) y1(i) = 0.00001

c870    continue


        imax = 0
        emax = 0.0

        do 501 i=2,ndata2

        if (e2(i).gt.emax) then
        imax = i
        emax = e2(i)
        endif

501     continue


        write (*,511)
        write (*,*) '________________________________________________'

        do 1522 k=imax-15,imax+10

        if (k.gt.1) then
        write (*,512) k,e2(k),y2(k)
        endif
1522    continue

        write (*,*) 'From which channel do you wish to start
     & your analysis for the excimer decay?'
        read(*,*) nstart2(3)

        write (*,*) 'At which channel does the background noise begin
     & for the excimer decay?'
        read(*,*) nstart2(1)

        write (*,*) 'At which channel does the background noise end
     & for the excimer decay?'
        read(*,*) nstart2(2)

c       sume = 0.0
c       sumy = 0.0
```

```
c      anback = nback2-nback1+1

c      do 861 i=nback1,nback2

c      sume = sume + e2(i)
c      sumy = sumy + y2(i)

c861   continue

c      sume = sume/anback
c      sumy = sumy/anback

c      do 871 i=1,ndata2

c      e2(i) = e2(i) - sume
c      y2(i) = y2(i) - sumy

c      if(e2(i).lt.0.0) e2(i) = 0.00001
c      if(y2(i).lt.0.0) y2(i) = 0.00001

c871   continue


       write (*,*) 'Give an estimate of the exchange rate constant.'
       read (*,*) akex

       write (*,*) 'Give an estimate of the average number of quencher
     & per blob.'
       read (*,*) an

       write (*,*) 'Give an estimate of the quenching rate constant
     & inside a blob.'
       read (*,*) akq

       a(2) = an*akq*akex/(akex+akq)
       a(3) = an*akq*akq/((akq+akex)*(akq+akex))
       a(4) = akq + akex

c      do 3 k=2,4
c      write (*,*) 'a(',k,') = ',a(k)
c3     continue

       write (*,*) 'What is the monomer lifetime?'
       read (*,*) tauM
```

```
        write (*,*) 'What is the pre-exponential factor of this
function?'
        read (*,*) a(1)

        write (*,*) 'What is your initial guess for the contribution of
     & unquenched ''free'' pyrene?'
        read (*,*) a(5)

        write (*,*) 'What is your initial guess for the contribution of
     & short-lived pyrene aggregates in the monomer decays Pyk2?'
        read (*,*) a(10)

        write (*,*) 'What is the rate constant for rapid excimer
formation
     & k2?'
        read (*,*) a(12)

        write (*,*) 'What is the contribution from dimers which form
excimer
     & instantaneously (E0*)?'
        read (*,*) a(7)

        a(8) = .1
        a(9) = .1

        a(15) = 10.0
        a(16) = 10.0

        write (*,*) 'What is your estimate for the lifetime of the
excimer?'
        read (*,*) a(11)

        write (*,*) 'What is its pre-exponential factor for the long-
lived
     &  dimers?'
        read (*,*) a(13)

        write (*,*) 'What is your estimate for the lifetime of the
long-lived
     & GS pyrene dimers?'
        read(*,*) a(14)

        write (*,*) 'What scaling factor do you wish to use to scale
the
     & parameters obtained during the fit of the monomer and excimer?'
        read (*,*) a(6)
```

```
       do 524 i=1,mfit
       lista(i) =i
524    continue

513    alambda = -0.1
       itera = 0
       kchi = 0

514    continue

       if (itera.ne.0) goto 540

515    write (*,521)
521    format(10Hiteration ,20H    amplitude       ,
     &20H    lifetime        ,20H    scattering      ,10H chisquare)
       write(*,*)'____________________________________________________
_
     ____________'

c      write (*,*) 'just before entering mrqmin. tauM = ',tauM

540    continue

       a(1) = sqrt(a(1))
       a(2) = sqrt(a(2))
       a(3) = sqrt(a(3))
       a(4) = sqrt(a(4))
       a(5) = sqrt(a(5))
       a(6) = sqrt(a(6))
       a(7) = sqrt(a(7))
       a(10) = sqrt(a(10))
       a(13) = sqrt(a(13))
       a(15) = sqrt(a(15))
       a(16) = sqrt(a(16))

c      write (*,*) 'Before entering mrqmin.'

c      do 2 k=1,ma
c      write (*,*) 'a(',k,') = ',a(k)
c2     continue

       call
mrqmin(tauM,y1,y2,ndata1,ndata2,a,ma,lista,mfit,covar,alpha,
     & nca,chisq,alambda,nstart1,nstart2,e1,e2,itera,da,beta
```

```
     &  ,dyda,tpc1,tpc2,nexp,niter,
     &  tid1,tidi1,ted1,tedi1,div0,div1,div2,aa0)


      a(1) = a(1)*a(1)
      a(2) = a(2)*a(2)
      a(3) = a(3)*a(3)
      a(4) = a(4)*a(4)
      a(5) = a(5)*a(5)
      a(6) = a(6)*a(6)
      a(7) = a(7)*a(7)
      a(10) = a(10)*a(10)
      a(13) = a(13)*a(13)
      a(15) = a(15)*a(15)
      a(16) = a(16)*a(16)

      write (*,*)

      write (*,542) itera,a(1),a(10),a(5),a(8),
     & chisq/(ndata1+ndata2-nstart1(3)-nstart2(3)
     & +(nstart1(2)-nstart1(1))+(nstart2(2)-nstart2(1))-mfit)
542   format(I5,5H     ,4(3H   ,F10.3,7H       ),F10.3)

      write (*,543) a(15),a(16)
543   format(10HBackground,2(3H   ,F10.3,7H       ))

      akex = a(3)*a(4)*a(4)/(a(2) + a(3)*a(4))
      akq = a(2)*a(4)/(a(2) + a(3)*a(4))
      an = (a(2)+a(3)*a(4))*(a(2)+a(3)*a(4))/(a(3)*a(4)*a(4))

      write (*,522) akex,an,akq
522   format(10H          ,3(3H   ,F10.3,7H       ))

      write (*,522) a(10),a(12),a(9)
      write (*,522) a(7),a(6),a(11)
      write (*,522) a(13),a(6),a(14)
523   format(10H          ,2(3H   ,F10.3,7H       ))


c     write(*,*) 'alambda = ',alambda

      if (itera.eq.100) then
      goto 550
      endif
```

```
        if (itera.eq.1) goto 514
        if(chicca.eq.chisq) kchi = kchi+1
        if (chicca.ne.chisq) chicca = chisq
        if(chicca.ne.chisq) kchi = 0
        if (kchi.gt.50) goto 550
        goto 514

550     continue

c550    write (*,*) 'Do you want to try new amplitudes and lifetimes
c      & or starting analysis channel?'
c       write (*,*) 'yes = 1'
c       read(*,*) nn
c       if (nn.eq.1) then

c       do 525 i=1,nexp
c               ii = nexp+i
c               write (*,*) 'what is your new ',i,'th lifetime?'
c               read(*,*) a(ii)
c               write (*,*) 'what is your new ',i,'th amplitude?'
c               read(*,*) a(i)
c525    continue

c       write (*,*) 'what is your new scattering factor correction?'
c       read(*,*) a(mfit)

c               write (*,*) 'what is your new starting analysis
channel?'
c               read(*,*) nstart
c               goto 513
c       else
                write (*,*) 'this is the end, my friend!'
c       endif


        aE(1) = a(6)
        tauE(1) = a(11)

        do 1110 kiki=1,niter

        akiki = kiki-1

        div0(kiki) = 1.0/(a(2)+akiki*a(4)-a(12))
        aa0(kiki) = a(2)+akiki*a(4)

        do 1115 ka=1,nexp
```

```
      div2(kiki,ka) = 1.0/(a(2)+akiki*a(4)+1.0/tauM-1.0/tauE(ka))

1115  continue

      do 1114 ka=1,nexp

      div1(ka) = 1.0/(a(12)+1.0/tauM-1.0/tauE(ka))

1114  continue


1110  continue


      a(1) = sqrt(a(1))
      a(2) = sqrt(a(2))
      a(3) = sqrt(a(3))
      a(4) = sqrt(a(4))
      a(5) = sqrt(a(5))
      a(6) = sqrt(a(6))
      a(7) = sqrt(a(7))
      a(10) = sqrt(a(10))
      a(13) = sqrt(a(13))
      a(15) = sqrt(a(15))
      a(16) = sqrt(a(16))

      do 1000 i=1,ndata1

      call foncs1(i,a,ymod,dyda,ma,ndata1,e1,tpc1,tauM,
     & nexp,niter,tid1,tidi1,ted1,tedi1,div0,div1,div2
     & ,aa0)

      ym(i) = ymod
      res1(i) = y1(i) - ymod

      if (ymod.lt.1.0) then

             res1(i) = 0.0

      else

             res1(i) = res1(i)/sqrt(ymod)

      endif
```

```
      if(i.lt.nstart1(3)) res1(i) = 0.0

1000  continue

      do 1010 i=1,ndata1

      sum = sum + res1(i)*res1(i)

1010  continue

      n3 = ndata1 - nstart1(3) + 1
      an3 = ndata1 - nstart1(3) + 1

      do 1020 j=nstart1(3),n3-1

             do 1030 i=nstart1(3),ndata1-j

             auto1(j) = auto1(j) + res1(i)*res1(i+j)

1030         continue

      am = j
      am = an3 - am

      if(am.eq.0.0) then

      write (*,*) 'there is a problem!'

      endif

      auto1(j) = an3*auto1(j)/(am*sum)

1020  continue

      open(2,file='plot1.dat',status='old')

      do 1040 i=1,ndata1

      time = i*tpc1
      write (2,1050) time,e1(i),y1(i),ym(i),res1(i),auto1(i)

1050  format (4F10.2,2E12.3)

1040  continue

      close(2)
```

```
      a(1) = a(1)*a(1)
      a(2) = a(2)*a(2)
      a(3) = a(3)*a(3)
      a(4) = a(4)*a(4)
      a(5) = a(5)*a(5)
      a(6) = a(6)*a(6)
      a(7) = a(7)*a(7)
      a(10) = a(10)*a(10)
      a(13) = a(13)*a(13)
      a(15) = a(15)*a(15)
      a(16) = a(16)*a(16)

      sum = a(1)+a(5)+a(10)

      akq = a(3)*a(4)*a(4)/(a(2) + a(3)*a(4))
      akex = a(2)*a(4)/(a(2) + a(3)*a(4))
      an = (a(2)+a(3)*a(4))*(a(2)+a(3)*a(4))/(a(3)*a(4)*a(4))


      open(2,file='plotres1',status='old')

      atcha = 0.0
      write(2,10) DD1
      write (2,1070) atcha,atcha
      write (2,1070) a(10)/sum,a(12)
      write (2,1070) a(1)/sum,akex
      write (2,1070) an,akq
      write (2,1070) a(5)/sum,tauM
      write (2,1080) a(15)
      write (2,1080) chisq/(ndata1+ndata2-nstart1(3)-nstart2(3) +
     & (nstart1(2)-nstart1(1))+(nstart2(2)-nstart2(1))-mfit)
      write (2,1075) nstart1(3)

1070  format(2F10.5)
1075  format (I5)
1080  format (F10.5)

      close(2)



      aE(1) = a(6)
      tauE(1) = a(11)

      do 1210 kiki=1,niter
```

```
        akiki = kiki-1

        div0(kiki) = 1.0/(a(2)+akiki*a(4)-a(12))
        aa0(kiki) = a(2)+akiki*a(4)

        do 1215 ka=1,nexp

        div2(kiki,ka) = 1.0/(a(2)+akiki*a(4)+1.0/tauM-1.0/tauE(ka))

1215    continue

        do 1214 ka=1,nexp

        div1(ka) = 1.0/(a(12)+1.0/tauM-1.0/tauE(ka))

1214    continue


1210    continue


        a(1) = sqrt(a(1))
        a(2) = sqrt(a(2))
        a(3) = sqrt(a(3))
        a(4) = sqrt(a(4))
        a(5) = sqrt(a(5))
        a(6) = sqrt(a(6))
        a(7) = sqrt(a(7))
        a(10) = sqrt(a(10))
        a(13) = sqrt(a(13))
        a(15) = sqrt(a(15))
        a(16) = sqrt(a(16))

        do 2000 i=1,ndata2

        call foncs2(i,a,ymod,dyda,ma,ndata2,e2,tpc2,tauM
      & ,nexp,niter,tid1,tidi1,ted1,tedi1,div0,div1,div2,aa0)

        ym(i) = ymod
        res2(i) = y2(i) - ymod

        if (ymod.lt.1.0) then

               res2(i) = 0.0
```

```
      else

             res2(i) = res2(i)/sqrt(ymod)

      endif

      if(i.lt.nstart2(3)) res2(i) = 0.0

2000  continue

      do 2010 i=1,ndata2

      sum = sum + res2(i)*res2(i)

2010  continue

      n3 = ndata2 - nstart2(3) + 1
      an3 = ndata2 - nstart2(3) + 1

      do 2020 j=nstart2(3),n3-1

             do 2030 i=nstart2(3),ndata2-j

             auto2(j) = auto2(j) + res2(i)*res2(i+j)

2030         continue

      am = j
      am = an3 - am

      if(am.eq.0.0) then

      write (*,*) 'there is a problem!'

      endif

      auto2(j) = an3*auto2(j)/(am*sum)

2020  continue

      open(2,file='plot2.dat',status='old')

      do 2040 i=1,ndata2

      time = i*tpc2
      write (2,1050) time,e2(i),y2(i),ym(i),res2(i),auto2(i)
```

```
2040   continue

       close(2)

       a(1) = a(1)*a(1)
       a(2) = a(2)*a(2)
       a(3) = a(3)*a(3)
       a(4) = a(4)*a(4)
       a(5) = a(5)*a(5)
       a(6) = a(6)*a(6)
       a(7) = a(7)*a(7)
       a(10) = a(10)*a(10)
       a(13) = a(13)*a(13)
       a(15) = a(15)*a(15)
       a(16) = a(16)*a(16)

       sum = a(1) + a(10) + a(7) + a(13)

       atcha = 0.0

       open(2,file='plotres2',status='old')

       atcha = 0.0
       write(2,10) 'globmis90gbg'
       write(2,10) DD2
       write (2,1070) a(1)/sum,tauM
       write (2,1070) a(10)/sum,a(12)
       write (2,1070) a(7)/sum,a(11)
       write (2,1070) a(13)/sum,a(14)
       write (2,1080) a(16)
       write (2,1080) chisq/(ndata1+ndata2-nstart1(3)-nstart2(3) +
     & (nstart1(2)-nstart1(1))+(nstart2(2)-nstart2(1))-mfit)
       write (2,1075) nstart2(3)

       close(2)


       end
```

```
      subroutine foncs1(i,a,ymod,dyda,ma,ndata1,e1,tpc1,tauM,
     & nexp,niter,tid1,tidi1,ted1,tedi1,div0,div1,div2,aa0)

      implicit double precision (a-h,o-z)

      real*8 a(ma),y1(2000),e1(2000),dyda(ma)
     &
,div0(niter),div1(nexp),div2(niter,nexp),aa0(niter),ted1(niter),
     & tedi1(niter),tid1(nexp),tidi1(nexp),aE(nexp),tauE(nexp)
      real*8 tadi1,tad1,amol,tpc1,
     & ymod,ymod0,
     & ymod1,ymod2,ymod3,ymod4,tauM,amol
     & ,ymod01,ymod02,ymod03,ymod04,ymod05


c     if (i.eq.1) then
c     write (*,*) 'We are in foncs1!'
c     write (*,*) 'tauM = ',tauM
c     endif

c     if (i.eq.1) then
c     write (*,*) 'in foncs1, tpc1 = ',tpc1
c     endif

      a(1) = a(1)*a(1)
      a(2) = a(2)*a(2)
      a(3) = a(3)*a(3)
      a(4) = a(4)*a(4)
      a(5) = a(5)*a(5)
      a(6) = a(6)*a(6)
      a(7) = a(7)*a(7)
      a(10) = a(10)*a(10)
      a(13) = a(13)*a(13)
      a(15) = a(15)*a(15)
      a(16) = a(16)*a(16)

      aE(1) = a(6)
      tauE(1) = a(11)


      do 1217 kiki=1,niter

      akiki = kiki-1

      ted1(kiki) = dexp(-(a(2)+akiki*a(4)+1.0/tauM)*tpc1)
      tedi1(kiki) = 1.0
```

```
1217    continue

c       if(i.eq.20) then
c       do 3 k=1,ma
c       write (*,*) 'a(',k,') = ',a(k)
c3      continue
c       endif

        do 5 k=1,ma
        dyda(k) = 0.
5       continue

        ymod0 = 0.0
        ymod1 = 0.0
        ymod2 = 0.0
        ymod3 = 0.0
        ymod4 = 0.0


        if (i.eq.1) goto 25

        tod1 = dexp(-(a(12)+1.0/tauM)*tpc1)
        todi1 = 1.0

        tud1 = dexp(-tpc1/tauM)
        tudi1 = 1.0

        tad1 = dexp(-a(4)*tpc1)
        tadi1 = 1.0

        do 20 k=1,i

        akk = k

        ymod01 = 0.0
        ymod02 = 0.0
        ymod03 = 0.0
        ymod04 = 0.0
        ymod05 = 0.0

        if((k.eq.1).or.(k.eq.i)) then
        amol = 0.5
        else
        amol = 1.0
        endif
```

```
  ymod0 = dexp( -(a(2)+1.0/tauM)*(akk-1.0)*tpc1 -
& a(3)*(1.0-tadi1) )
  ymod1 = ymod1 + amol*e1(i-k+1)*tpc1*a(1)*ymod0
  ymod2 = ymod2 + amol*e1(i-k+1)*tpc1*a(5)*tudi1
  ymod3 = ymod3 + amol*e1(i-k+1)*tpc1*a(10)*todi1


  dyda(2) = dyda(2) - amol*e1(i-k+1)*tpc1*a(1)*tpc1*(akk-1.0)
& *ymod0
  dyda(3) = dyda(3) - amol*e1(i-k+1)*tpc1*a(1)*
& (1.0-tadi1)*ymod0
  dyda(4) = dyda(4) - amol*e1(i-k+1)*tpc1*a(1)*tpc1*(akk-1.0)
& *tadi1*a(3)*ymod0

  ani = 1.0/a(3)
  faci = 1.0

  do 30 kk=1,niter

  aak = kk - 1
  ani = ani*a(3)

  ymod01 = ymod01 + ani*aa0(kk)*div0(kk)*
& (todi1-tedi1(kk))/faci

  ymod02 = ymod02 - ani*a(12)*div0(kk)*div0(kk)*
& (todi1 - tedi1(kk))/faci +
& ani*aa0(kk)*div0(kk)*(akk-1.0)*tpc1*tedi1(kk)/faci

  ymod03 = ymod03 - ani*aa0(kk)*div0(kk)*
& (todi1 - tedi1(kk))/faci +
& aak*ani*aa0(kk)*div0(kk)*
& (todi1-tedi1(kk))/(faci*a(3))

  ymod04 = ymod04 - ani*aak*a(12)*div0(kk)*div0(kk)*
& (todi1 - tedi1(kk))/faci +
& ani*aa0(kk)*aak*(akk-1.0)*tpc1*div0(kk)*
& tedi1(kk)/faci

  ymod05 = ymod05 + ani*aa0(kk)*div0(kk)*div0(kk)*
& todi1/faci -
& ani*aa0(kk)*div0(kk)*(akk-1.0)*tpc1*todi1/faci -
& ani*aa0(kk)*div0(kk)*div0(kk)*tedi1(kk)/faci

  faci = faci*(aak+1.0)
```

```
30      continue

        ymod4 = ymod4 + amol*e1(i-k+1)*tpc1*a(1)*dexp(-a(3))
     & *ymod01

        dyda(2) = dyda(2) + amol*e1(i-k+1)*tpc1*a(1)*dexp(-a(3))
     & *ymod02

        dyda(3) = dyda(3) + amol*e1(i-k+1)*tpc1*a(1)*dexp(-a(3))
     & *ymod03

        dyda(4) = dyda(4) + amol*e1(i-k+1)*tpc1*a(1)*dexp(-a(3))
     & *ymod04

        dyda(12) = dyda(12) + amol*e1(i-k+1)*tpc1*
     & (a(1)*dexp(-a(3))*ymod05 - a(10)*(akk-1.0)*tpc1*todi1)

        todi1 = todi1*tod1
        tudi1 = tudi1*tud1
        tadi1 = tadi1*tad1

        do 40 ketchup=1,niter
        tedi1(ketchup) = tedi1(ketchup)*ted1(ketchup)
40      continue

20      continue

25      continue

        dyda(1) = 2.0*sqrt(a(1))*(ymod1+ymod4)/a(1)
        dyda(2) = 2.0*sqrt(a(2))*dyda(2)
        dyda(3) = 2.0*sqrt(a(3))*dyda(3)
        dyda(4) = 2.0*sqrt(a(4))*dyda(4)
        dyda(5) = 2.0*sqrt(a(5))*ymod2/a(5)
        dyda(6) = 0.0
        dyda(7) = 0.0
        dyda(8) = e1(i)
        dyda(9) = 0.0
        dyda(10) = 2.0*sqrt(a(10))*ymod3/a(10)
        dyda(11) = 0.0
        dyda(13) = 0.0
        dyda(14) = 0.0
        dyda(15) = 2.0*sqrt(a(15))
        dyda(16) = 0.0
```

```
      ymod = ymod1 + ymod2 + ymod3 + ymod4 + a(8)*e1(i)
     & + a(15)

c     if (i.lt.10) then
c     write (*,*) 'in foncs1 ',i,e1(i),ymod
c     endif

c     write (*,*) dyda(1),dyda(2),dyda(3),dyda(4),dyda(5)

c     do 111 ik=1,ma
c111  write (*,*) 'a(',ik,' = ',a(ik)

      a(1) = sqrt(a(1))
      a(2) = sqrt(a(2))
      a(3) = sqrt(a(3))
      a(4) = sqrt(a(4))
      a(5) = sqrt(a(5))
      a(6) = sqrt(a(6))
      a(7) = sqrt(a(7))
      a(10) = sqrt(a(10))
      a(13) = sqrt(a(13))
      a(15) = sqrt(a(15))
      a(16) = sqrt(a(16))

      return

      end


      subroutine foncs2(i,a,ymod,dyda,ma,ndata2,e2,tpc2,tauM,
     & nexp,niter,tid1,tidi1,ted1,tedi1,div0,div1,div2,aa0)

      implicit double precision (a-h,o-z)

      real*8 a(ma),e2(2000),dyda(ma)
      real*8 amol,tpc2,ymod,ymod1(nexp),
     & ymod2(nexp),ymod3(nexp),ymod4(nexp),ymod5(nexp),tauM,aa0(niter)
     & ,tod1,todi1,ted1(niter),tedi1(niter),tid1(nexp),tidi1(nexp)
     & ,div0(niter),div1(nexp),div2(niter,nexp),aE(nexp),tauE(nexp)
     & ,akk,aak,ani,faci
     & ,ymod01(nexp),ymod02(nexp),ymod03(nexp),ymod04(nexp),
     & ymod05(nexp),ymod06(nexp),ymod07(nexp)

      a(1) = a(1)*a(1)
      a(2) = a(2)*a(2)
      a(3) = a(3)*a(3)
```

```
      a(4) = a(4)*a(4)
      a(5) = a(5)*a(5)
      a(6) = a(6)*a(6)
      a(7) = a(7)*a(7)
      a(10) = a(10)*a(10)
      a(13) = a(13)*a(13)
      a(15) = a(15)*a(15)
      a(16) = a(16)*a(16)

      aE(1) = a(6)
      tauE(1) = a(11)

      do 1216 ka=1,nexp

      tid1(ka) = dexp(-tpc2/tauE(ka))
      tidi1(ka) = 1.0

1216  continue

      tud1 = dexp(-tpc2/a(14))
      tudi1 = 1.0

      do 1217 kiki=1,niter

      akiki = kiki-1

      ted1(kiki) = dexp(-(a(2)+akiki*a(4)+1.0/tauM)*tpc2)
      tedi1(kiki) = 1.0

1217  continue

c     if(i.eq.1) then
c     write (*,*) 'Before operation on a''s.'
c     do 2 k=1,ma
c     write (*,*) 'a(',k,') = ',a(k)
c2    continue
c     endif

      do 5 k=1,ma
      dyda(k) = 0.
5     continue


      ymod = 0.0
      ymodD = 0.0
```

```
      do 50 ka=1,nexp

      ymod1(ka) = 0.0
      ymod2(ka) = 0.0
      ymod3(ka) = 0.0
      ymod4(ka) = 0.0
      ymod5(ka) = 0.0

50    continue

      if (i.eq.1) goto 25

      tod1 = dexp(-(a(12)+1.0/tauM)*tpc2)
      todi1 = 1.0


      do 20 k=1,i

      akk = k

      if((k.eq.1).or.(k.eq.i)) then
      amol = 0.5
      else
      amol = 1.0
      endif

      ani = 1.0/a(3)
      faci = 1.0

      do 60 ka=1,nexp

      ymod01(ka) = 0.0
      ymod02(ka) = 0.0
      ymod03(ka) = 0.0
      ymod04(ka) = 0.0
      ymod05(ka) = 0.0
      ymod06(ka) = 0.0
      ymod07(ka) = 0.0

60    continue

      do 30 kk=1,niter

      aak = kk
      ani = ani*a(3)
```

```
c       write (*,*) kk,aak

c       if ((i.eq.2).and.(k.eq.1)) then
c       write (*,*) kk,ani
c       endif

        do 22 ka=1,nexp

        ymod01(ka) = ymod01(ka) +
     & ani*aa0(kk)*div0(kk)*div1(ka)*aE(ka)*
     & (-todi1+tidi1(ka))/faci -
     & ani*aa0(kk)*div0(kk)*div2(kk,ka)*aE(ka)*
     & (tidi1(ka)-tedi1(kk))/faci

        ymod02(ka) = ymod02(ka) -
     & ani*a(12)*div0(kk)*div0(kk)*div1(ka)*
     & aE(ka)*(tidi1(ka)-todi1)/faci +
     & ani*a(12)*div0(kk)*div0(kk)*div2(kk,ka)*aE(ka)*
     & (tidi1(ka)-tedi1(kk))/faci +
     & ani*aa0(kk)*div0(kk)*div2(kk,ka)*div2(kk,ka)*aE(ka)*
     & (tidi1(ka)-tedi1(kk))/faci -
     & ani*aa0(kk)*div0(kk)*div2(kk,ka)*aE(ka)*
     & (akk-1.0)*tpc2*tedi1(kk)/faci

        ymod03(ka) = ymod03(ka) -
     & ani*aa0(kk)*div0(kk)*div1(ka)*aE(ka)*
     & (tidi1(ka)-todi1)/faci +
     & (aak-1.0)*ani*aa0(kk)*div0(kk)*div1(ka)*aE(ka)*
     & (tidi1(ka)-todi1)/(faci*a(3)) +
     & ani*aa0(kk)*div0(kk)*div2(kk,ka)*aE(ka)*
     & (tidi1(ka)-tedi1(kk))/faci -
     & (aak-1.0)*ani*aa0(kk)*div0(kk)*div2(kk,ka)*aE(ka)*
     & (tidi1(ka)-tedi1(kk))/(faci*a(3))


        ymod04(ka) = ymod04(ka) -
     & ani*(aak-1.0)*a(12)*
     & div0(kk)*div0(kk)*div1(ka)*aE(ka)*
     & (tidi1(ka)-todi1)/faci +
     & ani*(aak-1.0)*a(12)*div0(kk)*div0(kk)*div2(kk,ka)*aE(ka)*
     & (tidi1(ka)-tedi1(kk))/faci +
     & ani*aa0(kk)*(aak-1.0)*div0(kk)*div2(kk,ka)*div2(kk,ka)*aE(ka)*
     & (tidi1(ka)-tedi1(kk))/faci -
     & ani*aa0(kk)*div0(kk)*div2(kk,ka)*(aak-1.0)*aE(ka)*
     & (akk-1.0)*tpc2*tedi1(kk)/faci
```

```
      ymod06(ka) = ymod06(ka) +
     & ani*aa0(kk)*div0(kk)*div1(ka)*
     & (akk-1.0)*tpc2*tidi1(ka)/(tauE(ka)*tauE(ka)*faci) -
     & ani*aa0(kk)*div0(kk)*div1(ka)*div1(ka)*
     & (tidi1(ka)-todi1)/(tauE(ka)*tauE(ka)*faci) -
     & ani*aa0(kk)*div0(kk)*div2(kk,ka)*
     & (akk-1.0)*tpc2*tidi1(ka)/(tauE(ka)*tauE(ka)*faci) +
     & ani*aa0(kk)*div0(kk)*div2(kk,ka)*div2(kk,ka)*
     & (tidi1(ka)-tedi1(kk))/(tauE(ka)*tauE(ka)*faci)

      ymod07(ka) = ymod07(ka) +
     & ani*aa0(kk)*div0(kk)*div0(kk)*div1(ka)*
     & (tidi1(ka)-todi1)/faci -
     & ani*aa0(kk)*div0(kk)*div1(ka)*div1(ka)*
     & (tidi1(ka)-todi1)/faci +
     & ani*aa0(kk)*div0(kk)*div1(ka)*
     & (akk-1.0)*tpc2*todi1/faci -
     & ani*aa0(kk)*div0(kk)*div0(kk)*div2(kk,ka)*
     & (tidi1(ka)-tedi1(kk))/faci

22    continue

      faci = faci*aak

30    continue

      do 70 ka=1,nexp

      ymod1(ka) = ymod1(ka) + amol*e2(i-k+1)*tpc2*a(1)*
     & a(12)*dexp(-a(3))*ymod01(ka)

      ymod2(ka) = ymod2(ka) + amol*e2(i-k+1)*tpc2*
     & a(7)*aE(ka)*tidi1(ka)

      ymod5(ka) = ymod5(ka) + amol*e2(i-k+1)*tpc2*a(10)*
     & a(12)*aE(ka)*div1(ka)*(tidi1(ka)-todi1)

      dyda(2) = dyda(2) + amol*e2(i-k+1)*tpc2*
     & a(12)*a(1)*dexp(-a(3))*ymod02(ka)

      dyda(3) = dyda(3) + amol*e2(i-k+1)*tpc2*
     & a(12)*a(1)*dexp(-a(3))*ymod03(ka)

      dyda(4) = dyda(4) + amol*e2(i-k+1)*tpc2*
     & a(12)*a(1)*dexp(-a(3))*ymod04(ka)
```

```
      dyda(12) = dyda(12) + amol*e2(i-k+1)*tpc2*
     & a(12)*aE(ka)*( a(1)*dexp(-a(3))*ymod07(ka) +
     & a(10)*(akk-1.0)*tpc2*todi1*div1(ka) -
     & a(10)*div1(ka)*div1(ka)*(tidi1(ka)-todi1) )

70    continue

      dyda(11) = dyda(11) + amol*e2(i-k+1)*tpc2*( a(12)*aE(1)
     & *a(1)*dexp(-a(3))*ymod06(1) + aE(1)*a(7)*(akk-1.0)*tpc2
     & *tidi1(1)/(tauE(1)*tauE(1)) +
     & a(12)*aE(1)*a(10)*
     & ((akk-1.0)*tpc2*tidi1(1)*div1(1) -
     & (tidi1(1)-todi1)*div1(1)*div1(1) )/(tauE(1)*tauE(1))  )

100   continue

      ymodD = ymodD + amol*e2(i-k+1)*tpc2*aE(1)*a(13)*tudi1

      dyda(14) = dyda(14) + amol*e2(i-k+1)*tpc2*aE(1)*a(13)*
     & (akk-1.0)*tpc2*tudi1

      do 40 ketchup=1,niter

      tedi1(ketchup) = tedi1(ketchup)*ted1(ketchup)

40    continue

      do 42 ka=1,nexp

      tidi1(ka) = tidi1(ka)*tid1(ka)

42    continue

      todi1 = todi1*tod1
      tudi1 = tudi1*tud1

20    continue

25    continue

      do 80 ka=1,nexp

      dyda(1) = dyda(1) + ymod1(ka)
      dyda(7) = dyda(7) + ymod2(ka)
      dyda(10) = dyda(10) + ymod5(ka)
```

```
80      continue

        dyda(6) = ymod1(1) + ymod2(1) + ymod5(1) + ymodD


        dyda(1) = 2.0*sqrt(a(1))*dyda(1)/a(1)
        dyda(2) = 2.0*sqrt(a(2))*dyda(2)
        dyda(3) = 2.0*sqrt(a(3))*dyda(3)
        dyda(4) = 2.0*sqrt(a(4))*dyda(4)
        dyda(5) = 0.0
        dyda(6) = 2.0*sqrt(aE(1))*dyda(6)/aE(1)
        dyda(7) = 2.0*sqrt(a(7))*dyda(7)/a(7)
        dyda(8) = 0.0
        dyda(9) = e2(i)
        dyda(10) = 2.0*sqrt(a(10))*dyda(10)/a(10)
        dyda(13) = 2.0*sqrt(a(13))*ymodD/a(13)
        dyda(14) = dyda(14)/(a(14)*a(14))
        dyda(15) = 0.0
        dyda(16) = 2.0*sqrt(a(16))

        do 90 ka=1,nexp

        ymod = ymod + ymod1(ka) + ymod2(ka) + ymod5(ka)

90      continue

        dyda(12) = dyda(12) + ymod/a(12)

        ymod = ymod + ymodD + a(9)*e2(i) + a(16)

c       if(i.lt.10) then
c       write (*,*) 'in foncs2 ',i,ymod
c       endif

c       write (*,*) i,ymod,ymod1,ymod2,ymod3

c       if(i.eq.100) then
c       do 100 kiki=1,ma
c       write (*,*) 'dyda(',kiki,') = ',dyda(kiki)
c100    continue
c       endif

c       do 111 ik=1,ma
c111    write (*,*) 'a(',ik,' = ',a(ik)

        a(1) = sqrt(a(1))
```

```
        a(2) = sqrt(a(2))
        a(3) = sqrt(a(3))
        a(4) = sqrt(a(4))
        a(5) = sqrt(a(5))
        a(6) = sqrt(a(6))
        a(7) = sqrt(a(7))
        a(10) = sqrt(a(10))
        a(13) = sqrt(a(13))
        a(15) = sqrt(a(15))
        a(16) = sqrt(a(16))


        return

        end









        subroutine mrqmin(tauM,y1,y2,ndata1,ndata2,a,ma,lista,mfit,
      & covar,alpha,nca,chisq,alamda,nstart1,nstart2,e1,e2,itera,
      & da,beta,dyda,tpc1,tpc2,nexp,niter
      & ,tid1,tidi1,ted1,tedi1,div0,div1,div2,aa0)


        implicit double precision (a-h,o-z)

        parameter (mmax=20)


        dimension
y1(ndata1),y2(ndata2),a(ma),lista(ma),e1(ndata1),e2(ndata2),
      &
        covar(mfit,mfit),alpha(mfit,mfit),atry(mmax),beta(mfit),da(mfit
)
      &  ,dyda(mfit),tid1(nexp),tidi1(nexp),ted1(niter),
      &
tedi1(niter),div0(niter),div1(nexp),div2(niter,nexp),aa0(niter)
      & ,nstart1(3),nstart2(3)
```

```
      real*8 tauM,chisq,alamda,tpc1,tpc2

      save ochisq

c     write (*,*) 'we are in mrqmin. tauM = ',tauM
c     write (*,*) 'we are in mrqmin. tpc2 = ',tpc2

      if (alamda.lt.0) then
            kk = mfit+1
            do 12 j=1,ma
                  ihit=0
                  do 11 k=1,mfit
                        if(lista(k).eq.j) ihit=ihit+1
11                continue
                  if(ihit.eq.0) then
                        lista(kk)=j
                        kk = kk+1
                  else if (ihit.gt.1) then
                        pause 'improper permutation in lista'
                  endif
12          continue
      if(kk.ne.(ma+1)) pause 'improper permutation in lista'
      alamda = 0.001

c     write (*,*) 'in mrqmin before entering mrqcof, the a''s equal:'
c     do 111 kiki=1,ma
c     write (*,*) 'a(',kiki,') = ',a(kiki)
c111  continue

      call
mrqcof(y1,y2,ndata1,ndata2,a,ma,lista,mfit,alpha,beta,nca,chisq
     & ,nstart1,nstart2,e1,e2,tpc1,tpc2,dyda,tauM,nexp,niter
     &  ,tid1,tidi1,ted1,tedi1,div0,div1,div2,aa0)

c     write(*,43) itera,a(1),a(5),chisq/(ndata1+ndata2-nstart1-
nstart2-mfit)
c43   format(I5,5H     ,3(3H   ,F10.3,7H       ))


      do 13 j=1,ma
            atry(j)=a(j)
13    continue
      endif
      ochisq = chisq
      itera = itera+1
```

```
      do 15 j=1,mfit
             do 14 k=1,mfit
                    covar(j,k)=alpha(j,k)
14           continue
      covar(j,j)=alpha(j,j)*(1.+alamda)
      da(j) = beta(j)
15    continue

c     write (*,*) 'Just before Gaussj.'

      call gaussj(covar,mfit,nca,da,1,1)

c     write (*,*) 'Just after Gaussj.'


      if(alamda.eq.0) then
             call covsrt(covar,nca,ma,lista,mfit)
             return
      endif
      do 16 j=1,mfit
             atry(lista(j)) = a(lista(j))+da(j)
16    continue


      call
mrqcof(y1,y2,ndata1,ndata2,atry,ma,lista,mfit,covar,da,nca,chisq
     &  ,nstart1,nstart2,e1,e2,tpc1,tpc2,dyda,tauM,nexp,niter,
     &  tid1,tidi1,ted1,tedi1,div0,div1,div2,aa0)


      if (chisq.lt.ochisq) then
      alamda = 0.1*alamda
      ochisq=chisq
      do 18 j=1,mfit
             do 17 k=1,mfit
                    alpha(j,k)=covar(j,k)
17           continue
      beta(j)=da(j)
      a(lista(j))=atry(lista(j))
18    continue
      else
      alamda = 10.*alamda
      chisq=ochisq
      endif
```

```
        return
        end


        subroutine mrqcof(y1,y2,ndata1,ndata2,a,ma,lista,mfit,
     & alpha,beta,nalp,chisq
     & ,nstart1,nstart2,e1,e2,tpc1,tpc2,dyda,tauM
     & ,nexp,niter,tid1,tidi1,ted1,tedi1,div0,div1,div2,aa0)

        implicit double precision (a-h,o-z)

        dimension y1(2000),y2(2000),alpha(nalp,nalp),beta(mfit),
     &  dyda(mfit),lista(mfit),a(ma),e1(2000),e2(2000),
     & tid1(nexp),tidi1(nexp),ted1(niter),tedi1(niter),div0(niter),
     & div1(nexp),div2(niter,nexp),aa0(niter)
     & ,aE(nexp),tauE(nexp),nstart1(3),nstart2(3)

        real*8 tauM,chisq,tpc1,tpc2

c       write (*,*) 'we are in mrqcof. tauM = ',tauM
c       write (*,*) 'we are in mrqcof. tpc2 = ',tpc2

        do 112 j=1,mfit
               do 111 k=1,j
               alpha(j,k) = 0.
111            continue
        beta(j) = 0.
112     continue
        chisq=0.

        a(1) = a(1)*a(1)
        a(2) = a(2)*a(2)
        a(3) = a(3)*a(3)
        a(4) = a(4)*a(4)
        a(5) = a(5)*a(5)
        a(6) = a(6)*a(6)
        a(7) = a(7)*a(7)
        a(10) = a(10)*a(10)
        a(13) = a(13)*a(13)
        a(15) = a(15)*a(15)
        a(16) = a(16)*a(16)

c       write (*,*) 'in mrqcof, before entering foncs1.'
c       do 222 kiki=1,ma
c       write (*,*) 'a(',kiki,') = ',a(kiki)
c222    continue
```

```
      aE(1) = a(6)
      tauE(1) = a(11)


      do 1110 kiki=1,niter

      akiki = kiki-1

      div0(kiki) = 1.0/(a(2)+akiki*a(4)-a(12))
      aa0(kiki) = a(2)+akiki*a(4)

      do 1115 ka=1,nexp

      div2(kiki,ka) = 1.0/(a(2)+akiki*a(4)+1.0/tauM-1.0/tauE(ka))

1115  continue


      do 1114 ka=1,nexp

      div1(ka) = 1.0/(a(12)+1.0/tauM-1.0/tauE(ka))

1114  continue


1110  continue

      a(1) = sqrt(a(1))
      a(2) = sqrt(a(2))
      a(3) = sqrt(a(3))
      a(4) = sqrt(a(4))
      a(5) = sqrt(a(5))
      a(6) = sqrt(a(6))
      a(7) = sqrt(a(7))
      a(10) = sqrt(a(10))
      a(13) = sqrt(a(13))
      a(15) = sqrt(a(15))
      a(16) = sqrt(a(16))

120   do 115 i=nstart1(3),ndata1

             call foncs1(i,a,ymod,dyda,ma,ndata1,e1,tpc1,tauM,
     & nexp,niter,tid1,tidi1,ted1,tedi1,div0,div1,div2,aa0)

c            if (i.lt.nstart1) goto 115
```

```
            sig2i = 1./y1(i)
            dy = y1(i)-ymod
            do 114 j=1,mfit
                  wt=dyda(lista(j))*sig2i
                        do 113 k=1,j
                        alpha(j,k)=alpha(j,k)+wt*dyda(lista(k))
113                     continue
            beta(j)=beta(j)+dy*wt
114         continue
      chisq=chisq+dy*dy*sig2i
115   continue

320   do 315 i=nstart1(1),nstart1(2)

            call foncs1(i,a,ymod,dyda,ma,ndata1,e1,tpc1,tauM,
     & nexp,niter,tid1,tidi1,ted1,tedi1,div0,div1,div2,aa0)

c           if (i.lt.nstart1) goto 115
            sig2i = 1./y1(i)
            dy = y1(i)-ymod
            do 314 j=1,mfit
                  wt=dyda(lista(j))*sig2i
                        do 313 k=1,j
                        alpha(j,k)=alpha(j,k)+wt*dyda(lista(k))
313                     continue
            beta(j)=beta(j)+dy*wt
314         continue
      chisq=chisq+dy*dy*sig2i
315   continue

c     write (*,*) 'Chisq value after monomer: ',chisq

      a(1) = a(1)*a(1)
      a(2) = a(2)*a(2)
      a(3) = a(3)*a(3)
      a(4) = a(4)*a(4)
      a(5) = a(5)*a(5)
      a(6) = a(6)*a(6)
      a(7) = a(7)*a(7)
      a(10) = a(10)*a(10)
      a(13) = a(13)*a(13)
      a(15) = a(15)*a(15)
      a(16) = a(16)*a(16)

      aE(1) = a(6)
      tauE(1) = a(11)
```

```
      do 1210 kiki=1,niter

      akiki = kiki-1

      div0(kiki) = 1.0/(a(2)+akiki*a(4)-a(12))
      aa0(kiki) = a(2)+akiki*a(4)

      do 1215 ka=1,nexp

      div2(kiki,ka) = 1.0/(a(2)+akiki*a(4)+1.0/tauM-1.0/tauE(ka))

1215  continue


      do 1214 ka=1,nexp

      div1(ka) = 1.0/(a(12)+1.0/tauM-1.0/tauE(ka))

1214  continue


1210  continue

      a(1) = sqrt(a(1))
      a(2) = sqrt(a(2))
      a(3) = sqrt(a(3))
      a(4) = sqrt(a(4))
      a(5) = sqrt(a(5))
      a(6) = sqrt(a(6))
      a(7) = sqrt(a(7))
      a(10) = sqrt(a(10))
      a(13) = sqrt(a(13))
      a(15) = sqrt(a(15))
      a(16) = sqrt(a(16))

220   do 215 i=nstart2(3),ndata2

             call foncs2(i,a,ymod,dyda,ma,ndata2,e2,tpc2,tauM,
     & nexp,niter,tid1,tidi1,ted1,tedi1,div0,div1,div2,aa0)

c            if (i.lt.nstart2) goto 215
             sig2i = 1./y2(i)
             dy = y2(i)-ymod
             do 214 j=1,mfit
```

```
                  wt=dyda(lista(j))*sig2i
                        do 213 k=1,j
                        alpha(j,k)=alpha(j,k)+wt*dyda(lista(k))
213                     continue
            beta(j)=beta(j)+dy*wt
214         continue
      chisq=chisq+dy*dy*sig2i
215   continue

420   do 415 i=nstart2(1),nstart2(2)

            call foncs2(i,a,ymod,dyda,ma,ndata2,e2,tpc2,tauM,
    & nexp,niter,tid1,tidi1,ted1,tedi1,div0,div1,div2,aa0)

c           if (i.lt.nstart2) goto 215
            sig2i = 1./y2(i)
            dy = y2(i)-ymod
            do 414 j=1,mfit
                  wt=dyda(lista(j))*sig2i
                        do 413 k=1,j
                        alpha(j,k)=alpha(j,k)+wt*dyda(lista(k))
413                     continue
            beta(j)=beta(j)+dy*wt
414         continue
      chisq=chisq+dy*dy*sig2i
415   continue

c     write (*,*) 'Chisq value after excimer: ',chisq


      do 117 j=2,mfit
            do 116 k=1,j-1
            alpha(k,j)=alpha(j,k)
116         continue
117   continue
      return
      end




      subroutine covsrt(covar,ncvm,ma,lista,mfit)

      implicit double precision (a-h,o-z)
```

```
      dimension covar(ncvm,ncvm),lista(mfit)

      do 212 j=1,ma-1
             do 211 i=j+1,ma
             covar(i,j) = 0.
211          continue
212   continue
      do 214 i=1,mfit-1
             do 213 j=i+1,mfit
                    if(lista(j).gt.lista(i)) then
                           covar(lista(j),lista(i))=covar(i,j)
                    else
                           covar(lista(i),lista(j))=covar(i,j)
                    endif
213          continue
214   continue
      swap=covar(1,1)
      do 215 j=1,ma
      covar(1,j) = covar(j,j)
      covar(j,j) = 0.
215   continue
      covar(lista(1),lista(1))=swap
      do 216 j=2,mfit
      covar(lista(j),lista(j))=covar(1,j)
216   continue
      do 218 j=2,ma
             do 217 i=1,j-1
             covar(i,j)=covar(j,i)
217   continue
218   continue
      return
      end




      subroutine gaussj(a,n,np,b,m,mp)

      implicit double precision (a-h,o-z)

      parameter (nmax=50)
      dimension a(np,np),b(np),ipiv(nmax),indxr(nmax),indxc(nmax)
```

```
      do 311 j=1,n
            ipiv(j) = 0
311   continue

      do 322 i=1,n
            big = 0.

            do 313 j=1,n

                  if (ipiv(j).ne.1) then

                  do 312 k=1,n

                  if(ipiv(k).eq.0) then

                        if (abs(a(j,k)).ge.big) then
                        big = abs(a(j,k))
                        irow = j
                        icol = k
                        endif

                        else if (ipiv(k).gt.1) then
                        pause 'singular matrix'
                        endif

312               continue

                  endif

313         continue

      ipiv(icol) = ipiv(icol) + 1

      if (irow.ne.icol) then

            do 314 l=1,n
                  dum = a(irow,l)
                  a(irow,l) = a(icol,l)
                  a(icol,l) = dum
314         continue

                  dum = b(irow)
                  b(irow) = b(icol)
                  b(icol) = dum
```

```
      endif

      indxr(i) = irow
      indxc(i) = icol

      if (a(icol,icol).eq.0)  pause 'singular matrix'

      pivinv = 1./a(icol,icol)

      a(icol,icol) = 1.

      do 316 l=1,n
             a(icol,l) = a(icol,l)*pivinv
316   continue

             b(icol) = b(icol)*pivinv

      do 321 ll=1,n
             if (ll.ne.icol) then

                    dum = a(ll,icol)
                    a(ll,icol) = 0.

                    do 318 l=1,n
                           a(ll,l) = a(ll,l) - a(icol,l)*dum
318                 continue

                           b(ll) = b(ll) - b(icol)*dum

             endif

321   continue

322   continue

      do 324 l=n,1,-1
             if (indxr(l).ne.indxc(l)) then

                    do 323 k=1,n
                           dum = a(k,indxr(l))
                           a(k,indxr(l)) = a(k,indxc(l))
                           a(k,indxc(l)) = dum
323                 continue

             endif
```

```
324     continue

        return
        end
```

D] Listing of the program *globmis90bbg*:

```
c       Program created on October 8th, 2007.

c       This program fits the monomer and excimer decays simultaneously
c       with the blob model equation.

c       Excimer formation occur sequentially. Two monomers diffuse with
c       the blob model equation, form a pyrene aggregate that rearranges
c       quickly with a rate constant k2 to form the excimer. k2 is fixed
c       in the analysis.

c       The excimer decay is fitted with the blob model equation + a
c       mono exponential function for excited ground-state dimers whose
c       pre-exponential factors and decaytimes are fitted in the analysis.

c       There is an extra lifetime for the excimer to account for
c       long-lived ground-state dimers.

        implicit double precision (a-h,o-z)

        parameter (mfit=15,ma=15,nca=15,nexp=1,niter=11)

        dimension y1(2000),lista(mfit),a(mfit),
     & covar(mfit,mfit),alpha(mfit,mfit),
     & e1(2000),da(mfit),beta(mfit)
     & ,dyda(mfit),ym(2000),res1(2000),auto1(2000)
     & ,e2(2000),y2(2000),res2(2000),auto2(2000)
     & ,tid1(nexp),tidi1(nexp),ted1(niter),tedi1(niter),
     & div0(niter),div1(nexp),div2(niter,nexp),aa0(niter)
     & ,aE(nexp),tauE(nexp),nstart1(3),nstart2(3)

        real*8 tauM,chisq,tpc1,tpc2


10      format(A)
        character *30 DD1,DD2


        write (*,*) 'How many chanels do you want to work with
     & for the monomer decay?'
        read (*,*) ndata1

c       write (*,*) 'What is the name of your lamp file ?'
c       read(*,10) DD

c       open (1,file=DD,status='old')
```

```
c       read(1,*) (e(i),i=1,ndata)

c       close(1)

c       write (*,*) 'what is the name of your fluorescence decay?'
c       read(*,10) DD

c       open(1,file=DD,status='old')

c       read(1,*) (y(i),i=1,ndata)

c       close(1)

c       write (*,*) 'What is your file''s name for the monomer?'
c       read(*,10) DD1

c       open(1,file=DD1,status='old')

c       do 15 i=1,ndata1

c       read(1,*) e1(i),y1(i)

c15     continue

c       close (1)

        write (*,*) 'What is your lamp''s filename for
     & the monomer decay?'
        read(*,10) DD1

        open(1,file=DD1,status='old')

        do 13 i=1,9
13      read(1,*)

        do 15 i=1,ndata1

        read(1,*) x,e1(i)

15      continue

        close (1)


        write (*,*) 'What is your monomer decay''s filename?'
```

```
        read(*,10) DD1

        open(1,file=DD1,status='old')

        do 14 i=1,9
14      read(1,*)

        do 16 i=1,ndata1

        read(1,*) x,y1(i)

16      continue

        close (1)


        write (*,*) 'How many chanels do you want to work with
     & for the excimer decay?'
        read (*,*) ndata2

c       write (*,*) 'What is your file''s name for the excimer?'
c       read(*,10) DD2

c       open(1,file=DD2,status='old')

c       do 16 i=1,ndata2

c       read(1,*) e2(i),y2(i)

c16     continue

c       close (1)


        write (*,*) 'What is your lamp''s filename for
     & the excimer decay?'
        read(*,10) DD2

        open(1,file=DD2,status='old')

        do 313 i=1,9
313     read(1,*)

        do 315 i=1,ndata2

        read(1,*) x,e2(i)
```

```
315     continue

        close (1)


        write (*,*) 'What is your excimer decay''s filename?'
        read(*,10) DD2

        open(1,file=DD2,status='old')

        do 314 i=1,9
314     read(1,*)

        do 316 i=1,ndata2

        read(1,*) x,y2(i)

316     continue

        close (1)


c       write (*,*) 'What is your lamp''s filename for the monomer?'
c       read(*,10) DD1

c       open(1,file=DD1,status='old')

c       read(1,*)

c       do 15 i=1,ndata1

c       read(1,*) e1(i)

c15     continue

c       close (1)

c       write (*,*) 'What is your decay''s filename for the monomer?'
c       read(*,10) DD1

c       open(1,file=DD1,status='old')

c       read(1,*)

c       do 16 i=1,ndata1
```

```
c       read(1,*) y1(i)

c16     continue

c       close (1)


c       write (*,*) 'What is your lamp''s filename for the excimer?'
c       read(*,10) DD2

c       open(1,file=DD2,status='old')

c       read(1,*)

c       do 315 i=1,ndata2

c       read(1,*) e2(i)

c315    continue

c       close (1)

c       write (*,*) 'What is your decay''s filename for the excimer?'
c       read(*,10) DD2

c       open(1,file=DD2,status='old')

c       read(1,*)

c       do 316 i=1,ndata2

c       read(1,*) y2(i)

c316    continue

c       close (1)

        write (*,*) 'What is the lifetime of the reference compound
     & for the monomer decays?'
        read(*,*) taur1

        write (*,*) 'What is your time per chanel for the monomer decays?'
        read(*,*) tpc1

        taur1 = exp(-tpc1/taur1)
```

```
      alold2 = e1(1)

      do 40 i=2,ndata1

      alold1 = e1(i)
      e1(i) = e1(i) - alold2*taur1
      alold2 = alold1

      if(e1(i).lt.0) e1(i)=0.0001

40    continue


      write (*,*) 'What is the lifetime of the reference compound
   & for the excimer decays?'
      read(*,*) taur2

      write (*,*) 'What is your time per chanel for the excimer decays?'
      read(*,*) tpc2

      taur2 = exp(-tpc2/taur2)

      alold2 = e2(1)

      do 41 i=2,ndata2

      alold1 = e2(i)
      e2(i) = e2(i) - alold2*taur2
      alold2 = alold1

      if(e2(i).lt.0) e2(i)=0.0001

41    continue

      imax = 0

      do 500 i=2,ndata1

      if (e1(i).gt.emax) then
      imax = i
      emax = e1(i)
      endif

500   continue
```

```
        write (*,511)
        write (*,*) '_______________________________________________________'
511     format(10H   channel,10H      lamp,10H     decay)

        do 520 k=imax-15,imax+10

        if (k.gt.1) then
        write (*,512) k,e1(k),y1(k)
512     format (3H   ,I4,3H   ,2(2H  ,F7.1,1H ))
        endif
520     continue

        write (*,*) 'From which channel do you wish to start
     &  your analysis for the monomer decay?'
        read(*,*) nstart1(3)

        write (*,*) 'At which channel does the background noise start
     & for the monomer decay?'
        read(*,*) nstart1(1)

        write (*,*) 'At which channel does the background noise end
     & for the monomer decay?'
        read(*,*) nstart1(2)

c       sume = 0.0
c       sumy = 0.0

c       anback = nback1

c       do 860 i=1,nback1

c       sume = sume + e1(i)
c       sumy = sumy + y1(i)

c860    continue

c       sume = sume/anback
c       sumy = sumy/anback

c       do 870 i=1,ndata1

c       e1(i) = e1(i) - sume
c       y1(i) = y1(i) - sumy

c       if(e1(i).lt.0.0) e1(i) = 0.00001
```

```
c       if(y1(i).lt.0.0) y1(i) = 0.00001

c870    continue


        imax = 0
        emax = 0.0

        do 501 i=2,ndata2

        if (e2(i).gt.emax) then
        imax = i
        emax = e2(i)
        endif

501     continue


        write (*,511)
        write (*,*) '_____________________________________________________'

        do 1522 k=imax-15,imax+10

        if (k.gt.1) then
        write (*,512) k,e2(k),y2(k)
        endif
1522    continue

        write (*,*) 'From which channel do you wish to start
     &  your analysis for the excimer decay?'
        read(*,*) nstart2(3)

        write (*,*) 'At which channel does the background noise start
     & for the excimer decay?'
        read(*,*) nstart2(1)

        write (*,*) 'At which channel does the background noise end
     & for the excimer decay?'
        read(*,*) nstart2(2)

c       sume = 0.0
c       sumy = 0.0

c       anback = nback2

c       do 861 i=1,nback2
```

```
c       sume = sume + e2(i)
c       sumy = sumy + y2(i)

c861    continue

c       sume = sume/anback
c       sumy = sumy/anback

c       do 871 i=1,ndata2

c       e2(i) = e2(i) - sume
c       y2(i) = y2(i) - sumy

c       if(e2(i).lt.0.0) e2(i) = 0.00001
c       if(y2(i).lt.0.0) y2(i) = 0.00001

c871    continue


        write (*,*) 'Give an estimate of the exchange rate constant.'
        read (*,*) akex

        write (*,*) 'Give an estimate of the average number of quencher
     & per blob.'
        read (*,*) an

        write (*,*) 'Give an estimate of the quenching rate constant
     & inside a blob.'
        read (*,*) akq

        a(2) = an*akq*akex/(akex+akq)
        a(3) = an*akq*akq/((akq+akex)*(akq+akex))
        a(4) = akq + akex

c       do 3 k=2,4
c       write (*,*) 'a(',k,') = ',a(k)
c3      continue

        write (*,*) 'What is the monomer lifetime?'
        read (*,*) tauM

        write (*,*) 'What is the pre-exponential factor of this function?'
        read (*,*) a(1)

        write (*,*) 'What is your initial guess for the contribution of
```

```
     & unquenched "free" pyrene?'
      read (*,*) a(5)

      write (*,*) 'What is your initial guess for the contribution of
     & short-lived pyrene aggregates in the monomer decays Pyk2?'
      read (*,*) a(10)

      write (*,*) 'What is the rate constant for rapid excimer formation
     & k2 (fixed in the analysis)?'
      read (*,*) ak2

      write (*,*) 'What is the contribution from dimers which form excimer
     & instantaneously (E0*)?'
      read (*,*) a(7)

      a(8) = .1
      a(9) = .1

      a(14) = 10.0
      a(15) = 10.0

      write (*,*) 'What is your estimate for the lifetime tauE?'
      read (*,*) a(12)
      write (*,*) 'What is the contribution from the long-lived dimers D*?'
      read (*,*) a(11)
      write (*,*) 'What is your estimate for the long lifetime tauD?'
      read (*,*) a(13)

      write (*,*) 'What scaling factor do you wish to use to scale the
     & parameters obtained during the fit of the monomer and excimer?'
      read (*,*) a(6)

      do 524 i=1,mfit
      lista(i) =i
524   continue

513   alambda = -0.1
      itera = 0
      kchi = 0

514   continue

      a(1) = sqrt(a(1))
      a(2) = sqrt(a(2))
      a(3) = sqrt(a(3))
      a(4) = sqrt(a(4))
```

```
      a(5) = sqrt(a(5))
      a(6) = sqrt(a(6))
      a(7) = sqrt(a(7))
      a(10) = sqrt(a(10))
      a(11) = sqrt(a(11))
      a(14) = sqrt(a(14))
      a(15) = sqrt(a(15))

      if (itera.ne.0) goto 540

515   write (*,521)
521   format(10Hiteration ,20H    amplitude       ,
   &20H    lifetime        ,20H    scattering      ,10H chisquare)
      write(*,*)'______________________________________________________
   _____________'

c     write (*,*) 'just before entering mrqmin. tauM = ',tauM

540   continue

c     write (*,*) 'Before entering mrqmin.'

c     do 2 k=1,ma
c     write (*,*) 'a(',k,') = ',a(k)
c2    continue

      call mrqmin(tauM,y1,y2,ndata1,ndata2,a,ma,lista,mfit,covar,alpha,
   &  nca,chisq,alambda,nstart1,nstart2,e1,e2,itera,da,beta
   & ,dyda,tpc1,tpc2,nexp,niter,
   & tid1,tidi1,ted1,tedi1,div0,div1,div2,aa0,ak2)


      a(1) = a(1)*a(1)
      a(2) = a(2)*a(2)
      a(3) = a(3)*a(3)
      a(4) = a(4)*a(4)
      a(5) = a(5)*a(5)
      a(6) = a(6)*a(6)
      a(7) = a(7)*a(7)
      a(10) = a(10)*a(10)
      a(11) = a(11)*a(11)
      a(14) = a(14)*a(14)
      a(15) = a(15)*a(15)

      write (*,*)
```

```
      write (*,542) itera,a(1),a(10),a(5),a(8),
     &   chisq/(ndata1+ndata2-nstart1(3)-nstart2(3)-
     & (nstart1(2)-nstart1(1))-(nstart2(2)-nstart2(1))-mfit)
542   format(I5,5H     ,4(3H   ,F10.3,7H       ),F10.3)

      write (*,543) a(14),a(15)
543   format(10HBackground,2(3H   ,F6.2,7H       ))

      akex = a(3)*a(4)*a(4)/(a(2) + a(3)*a(4))
      akq = a(2)*a(4)/(a(2) + a(3)*a(4))
      an = (a(2)+a(3)*a(4))*(a(2)+a(3)*a(4))/(a(3)*a(4)*a(4))

      write (*,522) akex,an,akq
522   format(10H          ,3(3H   ,F10.3,7H       ))

      write (*,523) tauM,a(9)
      write (*,522) a(6),a(7),a(12)
      write (*,522) a(6),a(11),a(13)
523   format(10H          ,2(3H   ,F10.3,7H       ))


c     write(*,*) 'alambda = ',alambda

      if (itera.eq.100) then
      goto 550
      endif


      if (itera.eq.1) goto 514
      if(chicca.eq.chisq) kchi = kchi+1
      if (chicca.ne.chisq) chicca = chisq
      if(chicca.ne.chisq) kchi = 0
      if (kchi.gt.50) goto 550
      goto 514

550   continue

c550  write (*,*) 'Do you want to try new amplitudes and lifetimes
c    & or starting analysis channel?'
c     write (*,*) 'yes = 1'
c     read(*,*) nn
c     if (nn.eq.1) then

c     do 525 i=1,nexp
c             ii = nexp+i
c             write (*,*) 'what is your new ',i,'th lifetime?'
```

```
c                 read(*,*) a(ii)
c                 write (*,*) 'what is your new ',i,'th amplitude?'
c                 read(*,*) a(i)
c525    continue

c       write (*,*) 'what is your new scattering factor correction?'
c       read(*,*) a(mfit)

c                 write (*,*) 'what is your new starting analysis channel?'
c                 read(*,*) nstart
c                 goto 513
c       else
                  write (*,*) 'this is the end, my friend!'
c       endif


        aE(1) = a(6)
        tauE(1) = a(12)

        do 1110 kiki=1,niter

        akiki = kiki-1

        div0(kiki) = 1.0/(a(2)+akiki*a(4)-ak2)
        aa0(kiki) = a(2)+akiki*a(4)

        do 1115 ka=1,nexp

        div2(kiki,ka) = 1.0/(a(2)+akiki*a(4)+1.0/tauM-1.0/tauE(ka))

1115    continue

        do 1114 ka=1,nexp

        div1(ka) = 1.0/(ak2+1.0/tauM-1.0/tauE(ka))

1114    continue


1110    continue


        a(1) = sqrt(a(1))
        a(2) = sqrt(a(2))
        a(3) = sqrt(a(3))
        a(4) = sqrt(a(4))
```

```
      a(5) = sqrt(a(5))
      a(6) = sqrt(a(6))
      a(7) = sqrt(a(7))
      a(10) = sqrt(a(10))
      a(11) = sqrt(a(11))
      a(14) = sqrt(a(14))
      a(15) = sqrt(a(15))

      do 1000 i=1,ndata1

      call foncs1(i,a,ymod,dyda,ma,ndata1,e1,tpc1,tauM,
     & nexp,niter,tid1,tidi1,ted1,tedi1,div0,div1,div2
     & ,aa0,ak2)

      ym(i) = ymod
      res1(i) = y1(i) - ymod

      if (ymod.lt.1.0) then

            res1(i) = 0.0

      else

            res1(i) = res1(i)/sqrt(ymod)

      endif

      if(i.lt.nstart1(3)) res1(i) = 0.0

1000  continue

      do 1010 i=1,ndata1

      sum = sum + res1(i)*res1(i)

1010  continue

      n3 = ndata1 - nstart1(3) + 1
      an3 = ndata1 - nstart1(3) + 1

      do 1020 j=nstart1(3),n3-1

            do 1030 i=nstart1(3),ndata1-j

            auto1(j) = auto1(j) + res1(i)*res1(i+j)
```

```
1030          continue

      am = j
      am = an3 - am

      if(am.eq.0.0) then

      write (*,*) 'there is a problem!'

      endif

      auto1(j) = an3*auto1(j)/(am*sum)

1020  continue

      open(2,file='plot1.dat',status='old')

      do 1040 i=1,ndata1

      time = i*tpc1
      write (2,1050) time,e1(i),y1(i),ym(i),res1(i),auto1(i)

1050  format (4F10.2,2E12.3)

1040  continue

      close(2)

      a(1) = a(1)*a(1)
      a(2) = a(2)*a(2)
      a(3) = a(3)*a(3)
      a(4) = a(4)*a(4)
      a(5) = a(5)*a(5)
      a(6) = a(6)*a(6)
      a(7) = a(7)*a(7)
      a(10) = a(10)*a(10)
      a(11) = a(11)*a(11)
      a(14) = a(14)*a(14)
      a(15) = a(15)*a(15)

      sum = a(1)+a(5)+a(10)

      akq = a(3)*a(4)*a(4)/(a(2) + a(3)*a(4))
      akex = a(2)*a(4)/(a(2) + a(3)*a(4))
      an = (a(2)+a(3)*a(4))*(a(2)+a(3)*a(4))/(a(3)*a(4)*a(4))
```

```
      open(2,file='plotres1',status='old')

      atcha = 0.0
      write(2,10) DD1
      write (2,1070) atcha,atcha
      write (2,1070) a(10)/sum,ak2
      write (2,1070) a(1)/sum,akex
      write (2,1070) an,akq
      write (2,1070) a(5)/sum,tauM
      write (2,1080) a(14)
      write (2,1080) chisq/(ndata1+ndata2-nstart1(3)-nstart2(3)-
   & (nstart1(2)-nstart1(1))-(nstart2(2)-nstart2(1))-mfit)
      write (2,1075) nstart1(3)

1070  format(2F10.5)
1075  format (I5)
1080  format (F10.5)

      close(2)



      aE(1) = a(6)
      tauE(1) = a(12)

      do 1210 kiki=1,niter

      akiki = kiki-1

      div0(kiki) = 1.0/(a(2)+akiki*a(4)-ak2)
      aa0(kiki) = a(2)+akiki*a(4)

      do 1215 ka=1,nexp

      div2(kiki,ka) = 1.0/(a(2)+akiki*a(4)+1.0/tauM-1.0/tauE(ka))

1215  continue

      do 1214 ka=1,nexp

      div1(ka) = 1.0/(ak2+1.0/tauM-1.0/tauE(ka))

1214  continue
```

```
1210    continue


        a(1) = sqrt(a(1))
        a(2) = sqrt(a(2))
        a(3) = sqrt(a(3))
        a(4) = sqrt(a(4))
        a(5) = sqrt(a(5))
        a(6) = sqrt(a(6))
        a(7) = sqrt(a(7))
        a(10) = sqrt(a(10))
        a(11) = sqrt(a(11))
        a(14) = sqrt(a(14))
        a(15) = sqrt(a(15))

        do 2000 i=1,ndata2

        call foncs2(i,a,ymod,dyda,ma,ndata2,e2,tpc2,tauM
     & ,nexp,niter,tid1,tidi1,ted1,tedi1,div0,div1,div2,aa0,ak2)

        ym(i) = ymod
        res2(i) = y2(i) - ymod

        if (ymod.lt.1.0) then

                res2(i) = 0.0

        else

                res2(i) = res2(i)/sqrt(ymod)

        endif

        if(i.lt.nstart2(3)) res2(i) = 0.0

2000    continue

        do 2010 i=1,ndata2

        sum = sum + res2(i)*res2(i)

2010    continue

        n3 = ndata2 - nstart2(3) + 1
        an3 = ndata2 - nstart2(3) + 1
```

```
      do 2020 j=nstart2(3),n3-1

            do 2030 i=nstart2(3),ndata2-j

            auto2(j) = auto2(j) + res2(i)*res2(i+j)

2030        continue

      am = j
      am = an3 - am

      if(am.eq.0.0) then

      write (*,*) 'there is a problem!'

      endif

      auto2(j) = an3*auto2(j)/(am*sum)

2020  continue

      open(2,file='plot2.dat',status='old')

      do 2040 i=1,ndata2

      time = i*tpc2
      write (2,1050) time,e2(i),y2(i),ym(i),res2(i),auto2(i)

2040  continue

      close(2)

      a(1) = a(1)*a(1)
      a(2) = a(2)*a(2)
      a(3) = a(3)*a(3)
      a(4) = a(4)*a(4)
      a(5) = a(5)*a(5)
      a(6) = a(6)*a(6)
      a(7) = a(7)*a(7)
      a(10) = a(10)*a(10)
      a(11) = a(11)*a(11)
      a(14) = a(14)*a(14)
      a(15) = a(15)*a(15)

      sum = a(1) + a(10) + a(7) + a(11)
```

```
      atcha = 0.0

      open(2,file='plotres2',status='old')

      atcha = 0.0
      write(2,10) 'globmis90bbg'
      write(2,10) DD2
      write (2,1070) a(1)/sum,tauM
      write (2,1070) a(10)/sum,ak2
      write (2,1070) a(7)/sum,a(12)
      write (2,1070) a(11)/sum,a(13)
      write (2,1080) a(15)
      write (2,1080) chisq/(ndata1+ndata2-nstart1(3)-nstart2(3)-
     & (nstart1(2)-nstart1(1))-(nstart2(2)-nstart2(1))-mfit)
      write (2,1075) nstart2(3)

      close(2)


      end






      subroutine foncs1(i,a,ymod,dyda,ma,ndata1,e1,tpc1,tauM,
     & nexp,niter,tid1,tidi1,ted1,tedi1,div0,div1,div2,aa0,ak2)

      implicit double precision (a-h,o-z)

      real*8 a(ma),y1(2000),e1(2000),dyda(ma)
     & ,div0(niter),div1(nexp),div2(niter,nexp),aa0(niter),ted1(niter),
     & tedi1(niter),tid1(nexp),tidi1(nexp),aE(nexp),tauE(nexp)
      real*8 tadi1,tad1,amol,tpc1,
     & ymod,ymod0,
     & ymod1,ymod2,ymod3,ymod4,tauM,amol
     & ,ymod01,ymod02,ymod03,ymod04,ak2


c     if (i.eq.1) then
c     write (*,*) 'We are in foncs1!'
c     write (*,*) 'tauM = ',tauM
c     endif
```

```
c       if (i.eq.1) then
c       write (*,*) 'in foncs1, tpc1 = ',tpc1
c       endif

        a(1) = a(1)*a(1)
        a(2) = a(2)*a(2)
        a(3) = a(3)*a(3)
        a(4) = a(4)*a(4)
        a(5) = a(5)*a(5)
        a(6) = a(6)*a(6)
        a(7) = a(7)*a(7)
        a(10) = a(10)*a(10)
        a(11) = a(11)*a(11)
        a(14) = a(14)*a(14)
        a(15) = a(15)*a(15)

        aE(1) = a(6)
        tauE(1) = a(12)


        do 1217 kiki=1,niter

        akiki = kiki-1

        ted1(kiki) = dexp(-(a(2)+akiki*a(4)+1.0/tauM)*tpc1)
        tedi1(kiki) = 1.0

1217    continue

c       if(i.eq.20) then
c       do 3 k=1,ma
c       write (*,*) 'a(',k,') = ',a(k)
c3      continue
c       endif

        do 5 k=1,ma
        dyda(k) = 0.
5       continue

        ymod0 = 0.0
        ymod1 = 0.0
        ymod2 = 0.0
        ymod3 = 0.0
        ymod4 = 0.0
```

```
      if (i.eq.1) goto 25

      tod1 = dexp(-(ak2+1.0/tauM)*tpc1)
      todi1 = 1.0

      tud1 = dexp(-tpc1/tauM)
      tudi1 = 1.0

      tad1 = dexp(-a(4)*tpc1)
      tadi1 = 1.0

      do 20 k=1,i

      akk = k

      ymod01 = 0.0
      ymod02 = 0.0
      ymod03 = 0.0
      ymod04 = 0.0

      if((k.eq.1).or.(k.eq.i)) then
      amol = 0.5
      else
      amol = 1.0
      endif

      ymod0 = dexp( -(a(2)+1.0/tauM)*(akk-1.0)*tpc1 -
&  a(3)*(1.0-tadi1) )
      ymod1 = ymod1 + amol*e1(i-k+1)*tpc1*a(1)*ymod0
      ymod2 = ymod2 + amol*e1(i-k+1)*tpc1*a(5)*tudi1
      ymod3 = ymod3 + amol*e1(i-k+1)*tpc1*a(10)*todi1


      dyda(2) = dyda(2) - amol*e1(i-k+1)*tpc1*a(1)*tpc1*(akk-1.0)
&  *ymod0
      dyda(3) = dyda(3) - amol*e1(i-k+1)*tpc1*a(1)*
&  (1.0-tadi1)*ymod0
      dyda(4) = dyda(4) - amol*e1(i-k+1)*tpc1*a(1)*tpc1*(akk-1.0)
&  *tadi1*a(3)*ymod0

      ani = 1.0/a(3)
      faci = 1.0

      do 30 kk=1,niter

      aak = kk - 1
```

```
      ani = ani*a(3)

      ymod01 = ymod01 + ani*aa0(kk)*div0(kk)*
   & (todi1-tedi1(kk))/faci

      ymod02 = ymod02 - ani*ak2*div0(kk)*div0(kk)*
   & (todi1 - tedi1(kk))/faci +
   & ani*aa0(kk)*div0(kk)*(akk-1.0)*tpc1*tedi1(kk)/faci

      ymod03 = ymod03 - ani*aa0(kk)*div0(kk)*
   & (todi1 - tedi1(kk))/faci +
   & aak*ani*aa0(kk)*div0(kk)*
   & (todi1-tedi1(kk))/(faci*a(3))

      ymod04 = ymod04 - ani*aak*ak2*div0(kk)*div0(kk)*
   & (todi1 - tedi1(kk))/faci +
   & ani*aa0(kk)*aak*(akk-1.0)*tpc1*div0(kk)*
   & tedi1(kk)/faci


      faci = faci*(aak+1.0)

30    continue

      todi1 = todi1*tod1
      tudi1 = tudi1*tud1
      tadi1 = tadi1*tad1

      do 40 ketchup=1,niter
      tedi1(ketchup) = tedi1(ketchup)*ted1(ketchup)
40    continue


      ymod4 = ymod4 + amol*e1(i-k+1)*tpc1*a(1)*dexp(-a(3))
   & *ymod01

      dyda(2) = dyda(2) + amol*e1(i-k+1)*tpc1*a(1)*dexp(-a(3))
   & *ymod02

      dyda(3) = dyda(3) + amol*e1(i-k+1)*tpc1*a(1)*dexp(-a(3))
   & *ymod03

      dyda(4) = dyda(4) + amol*e1(i-k+1)*tpc1*a(1)*dexp(-a(3))
   & *ymod04
```

```
20      continue

25      continue

        dyda(1) = 2.0*sqrt(a(1))*(ymod1+ymod4)/a(1)
        dyda(2) = 2.0*sqrt(a(2))*dyda(2)
        dyda(3) = 2.0*sqrt(a(3))*dyda(3)
        dyda(4) = 2.0*sqrt(a(4))*dyda(4)
        dyda(5) = 2.0*sqrt(a(5))*ymod2/a(5)
        dyda(6) = 0.0
        dyda(7) = 0.0
        dyda(8) = e1(i)
        dyda(9) = 0.0
        dyda(10) = 2.0*sqrt(a(10))*ymod3/a(10)
        dyda(11) = 0.0
        dyda(12) = 0.0
        dyda(13) = 0.0
        dyda(14) = 2.0*sqrt(a(14))
        dyda(15) = 0.0

        ymod = ymod1 + ymod2 + ymod3 + ymod4 + a(8)*e1(i)
     & + a(14)

c       if (i.lt.10) then
c       write (*,*) 'in foncs1 ',i,e1(i),ymod
c       endif

c       write (*,*) dyda(1),dyda(2),dyda(3),dyda(4),dyda(5)

c       do 111 ik=1,ma
c111    write (*,*) 'a(',ik,' = ',a(ik)

        a(1) = sqrt(a(1))
        a(2) = sqrt(a(2))
        a(3) = sqrt(a(3))
        a(4) = sqrt(a(4))
        a(5) = sqrt(a(5))
        a(6) = sqrt(a(6))
        a(7) = sqrt(a(7))
        a(10) = sqrt(a(10))
        a(11) = sqrt(a(11))
        a(14) = sqrt(a(14))
        a(15) = sqrt(a(15))

        return
```

```
      end


      subroutine foncs2(i,a,ymod,dyda,ma,ndata2,e2,tpc2,tauM,
     & nexp,niter,tid1,tidi1,ted1,tedi1,div0,div1,div2,aa0,ak2)

      implicit double precision (a-h,o-z)

      real*8 a(ma),e2(2000),dyda(ma)
      real*8 amol,tpc2,ymod,ymod10,ymod1(nexp),
     & ymod2(nexp),ymod3(nexp),ymod4(nexp),ymod5(nexp),tauM,aa0(niter)
     & ,tod1,todi1,ted1(niter),tedi1(niter),tid1(nexp),tidi1(nexp)
     & ,div0(niter),div1(nexp),div2(niter,nexp),aE(nexp),tauE(nexp)
     & ,akk,aak,ani,faci,ak2
     & ,ymod01(nexp),ymod02(nexp),ymod03(nexp),ymod04(nexp),
     & ymod05(nexp),ymod06(nexp)

      a(1) = a(1)*a(1)
      a(2) = a(2)*a(2)
      a(3) = a(3)*a(3)
      a(4) = a(4)*a(4)
      a(5) = a(5)*a(5)
      a(6) = a(6)*a(6)
      a(7) = a(7)*a(7)
      a(10) = a(10)*a(10)
      a(11) = a(11)*a(11)
      a(14) = a(14)*a(14)
      a(15) = a(15)*a(15)

      aE(1) = a(6)
      tauE(1) = a(12)

      do 1216 ka=1,nexp

      tid1(ka) = dexp(-tpc2/tauE(ka))
      tidi1(ka) = 1.0

1216  continue

      do 1217 kiki=1,niter

      akiki = kiki-1

      ted1(kiki) = dexp(-(a(2)+akiki*a(4)+1.0/tauM)*tpc2)
      tedi1(kiki) = 1.0
```

```
1217    continue

c       if(i.eq.1) then
c       write (*,*) 'Before operation on a''s.'
c       do 2 k=1,ma
c       write (*,*) 'a(',k,') = ',a(k)
c2      continue
c       endif

        do 5 k=1,ma
        dyda(k) = 0.
5       continue


        ymod = 0.0
        ymod10 = 0.0

        do 50 ka=1,nexp

        ymod1(ka) = 0.0
        ymod2(ka) = 0.0
        ymod3(ka) = 0.0
        ymod4(ka) = 0.0
        ymod5(ka) = 0.0

50      continue

        if (i.eq.1) goto 25

        tod1 = dexp(-(ak2+1.0/tauM)*tpc2)
        todi1 = 1.0

        tud1 = dexp(-tpc2/a(13))
        tudi1 = 1.0


        do 20 k=1,i

        akk = k

        if((k.eq.1).or.(k.eq.i)) then
        amol = 0.5
        else
        amol = 1.0
        endif
```

```
      ani = 1.0/a(3)
      faci = 1.0

      do 60 ka=1,nexp

      ymod01(ka) = 0.0
      ymod02(ka) = 0.0
      ymod03(ka) = 0.0
      ymod04(ka) = 0.0
      ymod05(ka) = 0.0
      ymod06(ka) = 0.0

60    continue

      do 30 kk=1,niter

      aak = kk
      ani = ani*a(3)

c     write (*,*) kk,aak

c     if ((i.eq.2).and.(k.eq.1)) then
c     write (*,*) kk,ani
c     endif

      do 22 ka=1,nexp

      ymod01(ka) = ymod01(ka) +
     & ani*aa0(kk)*div0(kk)*div1(ka)*aE(ka)*
     & (-todi1+tidi1(ka))/faci -
     & ani*aa0(kk)*div0(kk)*div2(kk,ka)*aE(ka)*
     & (tidi1(ka)-tedi1(kk))/faci

      ymod02(ka) = ymod02(ka) -
     & ani*ak2*div0(kk)*div0(kk)*div1(ka)*
     & aE(ka)*(tidi1(ka)-todi1)/faci +
     & ani*ak2*div0(kk)*div0(kk)*div2(kk,ka)*aE(ka)*
     & (tidi1(ka)-tedi1(kk))/faci +
     & ani*aa0(kk)*div0(kk)*div2(kk,ka)*div2(kk,ka)*aE(ka)*
     & (tidi1(ka)-tedi1(kk))/faci -
     & ani*aa0(kk)*div0(kk)*div2(kk,ka)*aE(ka)*
     & (akk-1.0)*tpc2*tedi1(kk)/faci

      ymod03(ka) = ymod03(ka) -
     & ani*aa0(kk)*div0(kk)*div1(ka)*aE(ka)*
     & (tidi1(ka)-todi1)/faci +
```

```
     & (aak-1.0)*ani*aa0(kk)*div0(kk)*div1(ka)*aE(ka)*
     & (tidi1(ka)-todi1)/(faci*a(3)) +
     & ani*aa0(kk)*div0(kk)*div2(kk,ka)*aE(ka)*
     & (tidi1(ka)-tedi1(kk))/faci -
     & (aak-1.0)*ani*aa0(kk)*div0(kk)*div2(kk,ka)*aE(ka)*
     & (tidi1(ka)-tedi1(kk))/(faci*a(3))


      ymod04(ka) = ymod04(ka) -
     & ani*(aak-1.0)*ak2*
     & div0(kk)*div0(kk)*div1(ka)*aE(ka)*
     & (tidi1(ka)-todi1)/faci +
     & ani*(aak-1.0)*ak2*div0(kk)*div0(kk)*div2(kk,ka)*aE(ka)*
     & (tidi1(ka)-tedi1(kk))/faci +
     & ani*aa0(kk)*(aak-1.0)*div0(kk)*div2(kk,ka)*div2(kk,ka)*aE(ka)*
     & (tidi1(ka)-tedi1(kk))/faci -
     & ani*aa0(kk)*div0(kk)*div2(kk,ka)*(aak-1.0)*aE(ka)*
     & (akk-1.0)*tpc2*tedi1(kk)/faci

      ymod06(ka) = ymod06(ka) +
     & ani*aa0(kk)*div0(kk)*div1(ka)*
     & (akk-1.0)*tpc2*tidi1(ka)/(tauE(ka)*tauE(ka)*faci) -
     & ani*aa0(kk)*div0(kk)*div1(ka)*div1(ka)*
     & (tidi1(ka)-todi1)/(tauE(ka)*tauE(ka)*faci) -
     & ani*aa0(kk)*div0(kk)*div2(kk,ka)*
     & (akk-1.0)*tpc2*tidi1(ka)/(tauE(ka)*tauE(ka)*faci) +
     & ani*aa0(kk)*div0(kk)*div2(kk,ka)*div2(kk,ka)*
     & (tidi1(ka)-tedi1(kk))/(tauE(ka)*tauE(ka)*faci)

22    continue

      faci = faci*aak

30    continue

      do 70 ka=1,nexp

      ymod1(ka) = ymod1(ka) + amol*e2(i-k+1)*tpc2*a(1)*
     & ak2*dexp(-a(3))*ymod01(ka)

      ymod2(ka) = ymod2(ka) + amol*e2(i-k+1)*tpc2*
     & a(7)*aE(ka)*tidi1(ka)

      ymod5(ka) = ymod5(ka) + amol*e2(i-k+1)*tpc2*a(10)*
     & ak2*aE(ka)*div1(ka)*(tidi1(ka)-todi1)
```

```
      dyda(2) = dyda(2) + amol*e2(i-k+1)*tpc2*
     & ak2*a(1)*dexp(-a(3))*ymod02(ka)
      dyda(3) = dyda(3) + amol*e2(i-k+1)*tpc2*
     & ak2*a(1)*dexp(-a(3))*ymod03(ka)
      dyda(4) = dyda(4) + amol*e2(i-k+1)*tpc2*
     & ak2*a(1)*dexp(-a(3))*ymod04(ka)

70    continue

      do 100 ka=1,nexp

      dyda(11+ka) = dyda(11+ka) + amol*e2(i-k+1)*tpc2*( ak2*aE(ka)
     & *a(1)*dexp(-a(3))*ymod06(ka) + aE(ka)*a(7)*(akk-1.0)*tpc2
     & *tidi1(ka)/(tauE(ka)*tauE(ka)) +
     & ak2*aE(ka)*a(10)*
     & ((akk-1.0)*tpc2*tidi1(ka)*div1(ka) -
     & (tidi1(ka)-todi1)*div1(ka)*div1(ka) )/(tauE(ka)*tauE(ka))  )

100   continue


      ymod10 = ymod10 + amol*e2(i-k+1)*tpc2*a(6)*a(11)*tudi1

      dyda(13) = dyda(13) + amol*e2(i-k+1)*tpc2*a(6)*a(11)*
     & tudi1*tpc2*(akk-1.0)/(a(13)*a(13))

      do 40 ketchup=1,niter

      tedi1(ketchup) = tedi1(ketchup)*ted1(ketchup)

40    continue

      do 42 ka=1,nexp

      tidi1(ka) = tidi1(ka)*tid1(ka)

42    continue

      todi1 = todi1*tod1
      tudi1 = tudi1*tud1

20    continue

25    continue

      do 80 ka=1,nexp
```

```
      dyda(1) = dyda(1) + ymod1(ka)
      dyda(7) = dyda(7) + ymod2(ka)
      dyda(10) = dyda(10) + ymod5(ka)

80    continue

      dyda(6) = ymod1(1) + ymod2(1) + ymod5(1) + ymod10


      dyda(1) = 2.0*sqrt(a(1))*dyda(1)/a(1)
      dyda(2) = 2.0*sqrt(a(2))*dyda(2)
      dyda(3) = 2.0*sqrt(a(3))*dyda(3)
      dyda(4) = 2.0*sqrt(a(4))*dyda(4)
      dyda(5) = 0.0
      dyda(6) = 2.0*sqrt(aE(1))*dyda(6)/aE(1)
      dyda(7) = 2.0*sqrt(a(7))*dyda(7)/a(7)
      dyda(8) = 0.0
      dyda(9) = e2(i)
      dyda(10) = 2.0*sqrt(a(10))*dyda(10)/a(10)
      dyda(11) = 2.0*sqrt(a(11))*ymod10/a(11)
      dyda(14) = 0.0
      dyda(15) = 2.0*sqrt(a(15))

      do 90 ka=1,nexp

      ymod = ymod + ymod1(ka) + ymod2(ka) + ymod5(ka)

90    continue

      ymod = ymod + ymod10 + a(9)*e2(i) + a(15)

c     if(i.lt.10) then
c     write (*,*) 'in foncs2 ',i,ymod
c     endif

c     write (*,*) i,ymod,ymod1,ymod2,ymod3

c     if(i.eq.100) then
c     do 100 kiki=1,ma
c     write (*,*) 'dyda(',kiki,') = ',dyda(kiki)
c100  continue
c     endif

c     do 111 ik=1,ma
c111  write (*,*) 'a(',ik,' = ',a(ik)
```

```
      a(1) = sqrt(a(1))
      a(2) = sqrt(a(2))
      a(3) = sqrt(a(3))
      a(4) = sqrt(a(4))
      a(5) = sqrt(a(5))
      a(6) = sqrt(a(6))
      a(7) = sqrt(a(7))
      a(10) = sqrt(a(10))
      a(11) = sqrt(a(11))
      a(14) = sqrt(a(14))
      a(15) = sqrt(a(15))


      return

      end











      subroutine mrqmin(tauM,y1,y2,ndata1,ndata2,a,ma,lista,mfit,
     & covar,alpha,nca,chisq,alamda,nstart1,nstart2,e1,e2,itera,
     & da,beta,dyda,tpc1,tpc2,nexp,niter
     & ,tid1,tidi1,ted1,tedi1,div0,div1,div2,aa0,ak2)


      implicit double precision (a-h,o-z)

      parameter (mmax=20)


      dimension y1(ndata1),y2(ndata2),a(ma),lista(ma),e1(ndata1),e2(ndata2),
     &  covar(mfit,mfit),alpha(mfit,mfit),atry(mmax),beta(mfit),da(mfit)
     &  ,dyda(mfit),tid1(nexp),tidi1(nexp),ted1(niter),
     &  tedi1(niter),div0(niter),div1(nexp),div2(niter,nexp),aa0(niter)
     &  ,nstart1(3),nstart2(3)

      real*8 tauM,chisq,alamda,tpc1,tpc2,ak2
```

```
      save ochisq

c     write (*,*) 'we are in mrqmin. tauM = ',tauM
c     write (*,*) 'we are in mrqmin. tpc2 = ',tpc2

      if (alamda.lt.0) then
            kk = mfit+1
            do 12 j=1,ma
                  ihit=0
                  do 11 k=1,mfit
                        if(lista(k).eq.j) ihit=ihit+1
11                continue
                  if(ihit.eq.0) then
                        lista(kk)=j
                        kk = kk+1
                  else if (ihit.gt.1) then
                        pause 'improper permutation in lista'
                  endif
12          continue
      if(kk.ne.(ma+1)) pause 'improper permutation in lista'
      alamda = 0.001

c     write (*,*) 'in mrqmin before entering mrqcof, the a''s equal:'
c     do 111 kiki=1,ma
c     write (*,*) 'a(',kiki,') = ',a(kiki)
c111  continue

      call mrqcof(y1,y2,ndata1,ndata2,a,ma,lista,mfit,alpha,beta,nca,chisq
   &  ,nstart1,nstart2,e1,e2,tpc1,tpc2,dyda,tauM,nexp,niter
   & ,tid1,tidi1,ted1,tedi1,div0,div1,div2,aa0,ak2)

c     write(*,43) itera,a(1),a(5),chisq/(ndata1+ndata2-nstart1-nstart2-mfit)
c43   format(I5,5H     ,3(3H   ,F10.3,7H       ))


      do 13 j=1,ma
            atry(j)=a(j)
13    continue
      endif
      ochisq = chisq
      itera = itera+1


      do 15 j=1,mfit
            do 14 k=1,mfit
```

```
                    covar(j,k)=alpha(j,k)
14              continue
        covar(j,j)=alpha(j,j)*(1.+alamda)
        da(j) = beta(j)
15      continue

c       write (*,*) 'Just before Gaussj.'

        call gaussj(covar,mfit,nca,da,1,1)

c       write (*,*) 'Just after Gaussj.'


        if(alamda.eq.0) then
                call covsrt(covar,nca,ma,lista,mfit)
                return
        endif
        do 16 j=1,mfit
                atry(lista(j)) = a(lista(j))+da(j)
16      continue


        call mrqcof(y1,y2,ndata1,ndata2,atry,ma,lista,mfit,covar,da,nca,chisq
     & ,nstart1,nstart2,e1,e2,tpc1,tpc2,dyda,tauM,nexp,niter,
     & tid1,tidi1,ted1,tedi1,div0,div1,div2,aa0,ak2)


        if (chisq.lt.ochisq) then
        alamda = 0.1*alamda
        ochisq=chisq
        do 18 j=1,mfit
                do 17 k=1,mfit
                    alpha(j,k)=covar(j,k)
17              continue
        beta(j)=da(j)
        a(lista(j))=atry(lista(j))
18      continue
        else
        alamda = 10.*alamda
        chisq=ochisq
        endif
        return
        end


        subroutine mrqcof(y1,y2,ndata1,ndata2,a,ma,lista,mfit,
```

```
     & alpha,beta,nalp,chisq
     &    ,nstart1,nstart2,e1,e2,tpc1,tpc2,dyda,tauM
     & ,nexp,niter,tid1,tidi1,ted1,tedi1,div0,div1,div2,aa0,ak2)

      implicit double precision (a-h,o-z)

      dimension y1(2000),y2(2000),alpha(nalp,nalp),beta(mfit),
     &  dyda(mfit),lista(mfit),a(ma),e1(2000),e2(2000),
     & tid1(nexp),tidi1(nexp),ted1(niter),tedi1(niter),div0(niter),
     & div1(nexp),div2(niter,nexp),aa0(niter)
     & ,aE(nexp),tauE(nexp),nstart1(3),nstart2(3)
      real*8 tauM,chisq,tpc1,tpc2,ak2

c     write (*,*) 'we are in mrqcof. tauM = ',tauM
c     write (*,*) 'we are in mrqcof. tpc2 = ',tpc2

      do 112 j=1,mfit
            do 111 k=1,j
            alpha(j,k) = 0.
111         continue
      beta(j) = 0.
112   continue
      chisq=0.

      a(1) = a(1)*a(1)
      a(2) = a(2)*a(2)
      a(3) = a(3)*a(3)
      a(4) = a(4)*a(4)
      a(5) = a(5)*a(5)
      a(6) = a(6)*a(6)
      a(7) = a(7)*a(7)
      a(10) = a(10)*a(10)
      a(11) = a(11)*a(11)
      a(14) = a(14)*a(14)
      a(15) = a(15)*a(15)

c     write (*,*) 'in mrqcof, before entering foncs1.'
c     do 222 kiki=1,ma
c     write (*,*) 'a(',kiki,') = ',a(kiki)
c222  continue

      aE(1) = a(6)
      tauE(1) = a(12)


      do 1110 kiki=1,niter
```

```
          akiki = kiki-1

          div0(kiki) = 1.0/(a(2)+akiki*a(4)-ak2)
          aa0(kiki) = a(2)+akiki*a(4)

          do 1115 ka=1,nexp

          div2(kiki,ka) = 1.0/(a(2)+akiki*a(4)+1.0/tauM-1.0/tauE(ka))

1115      continue


          do 1114 ka=1,nexp

          div1(ka) = 1.0/(ak2+1.0/tauM-1.0/tauE(ka))

1114      continue


1110      continue

          a(1) = sqrt(a(1))
          a(2) = sqrt(a(2))
          a(3) = sqrt(a(3))
          a(4) = sqrt(a(4))
          a(5) = sqrt(a(5))
          a(6) = sqrt(a(6))
          a(7) = sqrt(a(7))
          a(10) = sqrt(a(10))
          a(11) = sqrt(a(11))
          a(14) = sqrt(a(14))
          a(15) = sqrt(a(15))

120       do 115 i=nstart1(3),ndata1

                    call foncs1(i,a,ymod,dyda,ma,ndata1,e1,tpc1,tauM,
     & nexp,niter,tid1,tidi1,ted1,tedi1,div0,div1,div2,aa0,ak2)

c                   if (i.lt.nstart1) goto 115
                    sig2i = 1./y1(i)
                    dy = y1(i)-ymod
                    do 114 j=1,mfit
                              wt=dyda(lista(j))*sig2i
                                        do 113 k=1,j
                                        alpha(j,k)=alpha(j,k)+wt*dyda(lista(k))
```

```
113                             continue
               beta(j)=beta(j)+dy*wt
114            continue
        chisq=chisq+dy*dy*sig2i
115     continue


        do 315 i=nstart1(1),nstart1(2)

               call foncs1(i,a,ymod,dyda,ma,ndata1,e1,tpc1,tauM,
     & nexp,niter,tid1,tidi1,ted1,tedi1,div0,div1,div2,aa0,ak2)

c              if (i.lt.nstart1) goto 115
               sig2i = 1./y1(i)
               dy = y1(i)-ymod
               do 314 j=1,mfit
                      wt=dyda(lista(j))*sig2i
                             do 313 k=1,j
                             alpha(j,k)=alpha(j,k)+wt*dyda(lista(k))
313                          continue
               beta(j)=beta(j)+dy*wt
314            continue
        chisq=chisq+dy*dy*sig2i
315     continue

c       write (*,*) 'Chisq value after monomer: ',chisq

        a(1) = a(1)*a(1)
        a(2) = a(2)*a(2)
        a(3) = a(3)*a(3)
        a(4) = a(4)*a(4)
        a(5) = a(5)*a(5)
        a(6) = a(6)*a(6)
        a(7) = a(7)*a(7)
        a(10) = a(10)*a(10)
        a(11) = a(11)*a(11)
        a(14) = a(14)*a(14)
        a(15) = a(15)*a(15)

        aE(1) = a(6)
        tauE(1) = a(12)


        do 1210 kiki=1,niter

        akiki = kiki-1
```

```
      div0(kiki) = 1.0/(a(2)+akiki*a(4)-ak2)
      aa0(kiki) = a(2)+akiki*a(4)

      do 1215 ka=1,nexp

      div2(kiki,ka) = 1.0/(a(2)+akiki*a(4)+1.0/tauM-1.0/tauE(ka))

1215  continue


      do 1214 ka=1,nexp

      div1(ka) = 1.0/(ak2+1.0/tauM-1.0/tauE(ka))

1214  continue


1210  continue

      a(1) = sqrt(a(1))
      a(2) = sqrt(a(2))
      a(3) = sqrt(a(3))
      a(4) = sqrt(a(4))
      a(5) = sqrt(a(5))
      a(6) = sqrt(a(6))
      a(7) = sqrt(a(7))
      a(10) = sqrt(a(10))
      a(11) = sqrt(a(11))
      a(14) = sqrt(a(14))
      a(15) = sqrt(a(15))

220   do 215 i=nstart2(3),ndata2

            call foncs2(i,a,ymod,dyda,ma,ndata2,e2,tpc2,tauM,
     &      nexp,niter,tid1,tidi1,ted1,tedi1,div0,div1,div2,aa0,ak2)

c           if (i.lt.nstart2) goto 215
            sig2i = 1./y2(i)
            dy = y2(i)-ymod
            do 214 j=1,mfit
                  wt=dyda(lista(j))*sig2i
                        do 213 k=1,j
                        alpha(j,k)=alpha(j,k)+wt*dyda(lista(k))
213                     continue
            beta(j)=beta(j)+dy*wt
```

```
214             continue
        chisq=chisq+dy*dy*sig2i
215     continue

        do 415 i=nstart2(1),nstart2(2)

                call foncs2(i,a,ymod,dyda,ma,ndata2,e2,tpc2,tauM,
     &  nexp,niter,tid1,tidi1,ted1,tedi1,div0,div1,div2,aa0,ak2)

c               if (i.lt.nstart2) goto 215
                sig2i = 1./y2(i)
                dy = y2(i)-ymod
                do 414 j=1,mfit
                        wt=dyda(lista(j))*sig2i
                                do 413 k=1,j
                                alpha(j,k)=alpha(j,k)+wt*dyda(lista(k))
413                             continue
                beta(j)=beta(j)+dy*wt
414             continue
        chisq=chisq+dy*dy*sig2i
415     continue

c       write (*,*) 'Chisq value after excimer: ',chisq


        do 117 j=2,mfit
                do 116 k=1,j-1
                alpha(k,j)=alpha(j,k)
116             continue
117     continue
        return
        end





        subroutine covsrt(covar,ncvm,ma,lista,mfit)

        implicit double precision (a-h,o-z)

        dimension covar(ncvm,ncvm),lista(mfit)

        do 212 j=1,ma-1
                do 211 i=j+1,ma
```

```
                covar(i,j) = 0.
211             continue
212     continue
        do 214 i=1,mfit-1
                do 213 j=i+1,mfit
                        if(lista(j).gt.lista(i)) then
                                covar(lista(j),lista(i))=covar(i,j)
                        else
                                covar(lista(i),lista(j))=covar(i,j)
                        endif
213             continue
214     continue
        swap=covar(1,1)
        do 215 j=1,ma
        covar(1,j) = covar(j,j)
        covar(j,j) = 0.
215     continue
        covar(lista(1),lista(1))=swap
        do 216 j=2,mfit
        covar(lista(j),lista(j))=covar(1,j)
216     continue
        do 218 j=2,ma
                do 217 i=1,j-1
                covar(i,j)=covar(j,i)
217     continue
218     continue
        return
        end




        subroutine gaussj(a,n,np,b,m,mp)

        implicit double precision (a-h,o-z)

        parameter (nmax=50)
        dimension a(np,np),b(np),ipiv(nmax),indxr(nmax),indxc(nmax)

        do 311 j=1,n
                ipiv(j) = 0
311     continue
```

```
        do 322 i=1,n
                big = 0.

                do 313 j=1,n

                        if (ipiv(j).ne.1) then

                        do 312 k=1,n

                        if(ipiv(k).eq.0) then

                                if (abs(a(j,k)).ge.big) then
                                big = abs(a(j,k))
                                irow = j
                                icol = k
                                endif

                                else if (ipiv(k).gt.1) then
                                pause 'singular matrix'
                                endif

312                     continue

                        endif

313             continue

        ipiv(icol) = ipiv(icol) + 1

        if (irow.ne.icol) then

                do 314 l=1,n
                        dum = a(irow,l)
                        a(irow,l) = a(icol,l)
                        a(icol,l) = dum
314             continue

                        dum = b(irow)
                        b(irow) = b(icol)
                        b(icol) = dum

        endif

        indxr(i) = irow
        indxc(i) = icol
```

```
      if (a(icol,icol).eq.0)  pause 'singular matrix'

      pivinv = 1./a(icol,icol)

      a(icol,icol) = 1.

      do 316 l=1,n
            a(icol,l) = a(icol,l)*pivinv
316   continue

            b(icol) = b(icol)*pivinv

      do 321 ll=1,n
            if (ll.ne.icol) then

                  dum = a(ll,icol)
                  a(ll,icol) = 0.

                  do 318 l=1,n
                        a(ll,l) = a(ll,l) - a(icol,l)*dum
318               continue

                        b(ll) = b(ll) - b(icol)*dum

            endif

321   continue

322   continue

      do 324 l=n,1,-1
            if (indxr(l).ne.indxc(l)) then

                  do 323 k=1,n
                        dum = a(k,indxr(l))
                        a(k,indxr(l)) = a(k,indxc(l))
                        a(k,indxc(l)) = dum
323               continue

            endif

324   continue

      return
      end
```
